# Floral evolution by simplification in Monanthotaxis (Annonaceae) and hypotheses for pollination system shifts

**DOI:** 10.1038/s41598-018-30607-2

**Published:** 2018-08-13

**Authors:** Paul H. Hoekstra, Jan J. Wieringa, Erik Smets, Lars W. Chatrou

**Affiliations:** 1 Naturalis Biodiversity Center, National Herbarium of the Netherlands, Darwinweg 2, 2300 RA Leiden, The Netherlands; 2 Wageningen University & Research, Biosystematics Group, Droevendaalsesteeg 1, 6708 PB Wageningen, The Netherlands; 3 Ecology, Evolution and Biodiversity Conservation Section, KU Leuven, Kasteelpark Arenberg 31, box 2435, 3001 Leuven, Belgium

## Abstract

Simplification by reduction has occurred many times independently in the floral evolution of angiosperms. These reductions have often been attributed to changes in reproductive biology. In the angiosperm plant family Annonaceae, most species have flowers with six petals, and many stamens and carpels. In the genus *Monanthotaxis* several deviations from this pattern have been observed, including flowers that contain three petals and three stamens only. New DNA sequences were generated for 42 specimens of *Monanthotaxis*. Five chloroplast markers and two nuclear markers for 72 out of 94 species of *Monanthotaxis* were used to reconstruct a phylogeny of the genus, which revealed several well-supported, morphologically distinct clades. The evolution of four quantitative and two qualitative floral characters was mapped onto this phylogeny, demonstrating a reduction in flower size and number of flower parts in *Monanthotaxis*. A large variation in stamen forms and numbers, strong correlations between petal size, stamen and carpel number, combined with a non-gradual mode of evolution and the sympatric co-occurrence of *Monanthotaxis* species from different clades suggest that the high diversity in the African rainforest of this genus is caused by switches in pollination systems.

## Introduction

The evolution of life shows a trend towards increasing complexity and synorganisation. Concurrently, there is an evolutionary trend across the tree of life towards the loss of biological complexity by reduction^1^. Examples of such simplifications in evolution are independent losses of multicellularity in a variety of fungal lineages, losses of Hox genes across different animal groups, the reduction in the complexity and size of the gametophytic generation in land plants and genome reduction in parasitic plants^1–4^. Simplification also occurs widely in the evolution of flowers across the angiosperms. Despite some uncertainty in the inference of ancestral floral characters at the crown node of angiosperms, e.g regarding the number of perianth and stamen whorls, it is evident that reductive trends are widespread in the evolution of angiosperms^5^.

Some of these trends are evidently linked to fundamental changes in reproductive biology, such as the change from bi- to unisexual flowers with a concomitant reduction in number, or entire disappearance, of either stamens or carpels^6^. Many independent reductions in flower size are correlated with changes in pollination regime. Shifts from insect pollination towards either wind pollination or self-pollination are associated with a decrease of floral complexity. The evolutionary scale of such changes ranges from local variation within a species to synapomorphies that characterize major clades^7–9^. The presence of sterile stamens (staminodes) is frequently an intermediate step in the reduction of the androecium. Staminodes that do not obtain a new function, such as pollinator attractant, are often quickly lost during evolution. As a consequence, non-functional staminodes are generally only found in taxa where the reduction has occurred recently^10^.

Before the era of molecular phylogenetics one of the hypotheses on the origin of angiosperms was the euanthial theory, arguing that the ancestral angiosperm flowers contained many spirally arranged parts and subsequent reduction has taken place^11^. With the rise of molecular phylogenetics and the discoveries of fossil flowers from the Cretaceous^12^ the current leading hypothesis is that the ancestral angiosperm flowers had more than two perianth and stamen whorls^5^. Both increases in size and complexity, as well as simplification occurred later in evolution and can now be observed in extant angiosperms^12,13^. A reduction in the number of whorls of tepals and stamens has occurred in most modern angiosperms. The ancestral state of many other floral characters is uncertain for angiosperms, which is likely caused by the variability of many of those states in the early diverging angiosperms, complicating the inference of ancestral states^13^.

Among early diverging angiosperms, the Annonaceae are second in size after the Lauraceae, containing ca 2430 species^14^, and are amongst the dominant plant families in tropical forests worldwide^15^. The flowers of most species of Annonaceae contain three perianth whorls, viz. a single whorl of three sepals and two whorls of three petals. In the majority of species the flowers have a large number of stamens in multiple whorls, with the whorled pattern often becoming irregular with increasing numbers of stamens^16^.

**Figure 1:**
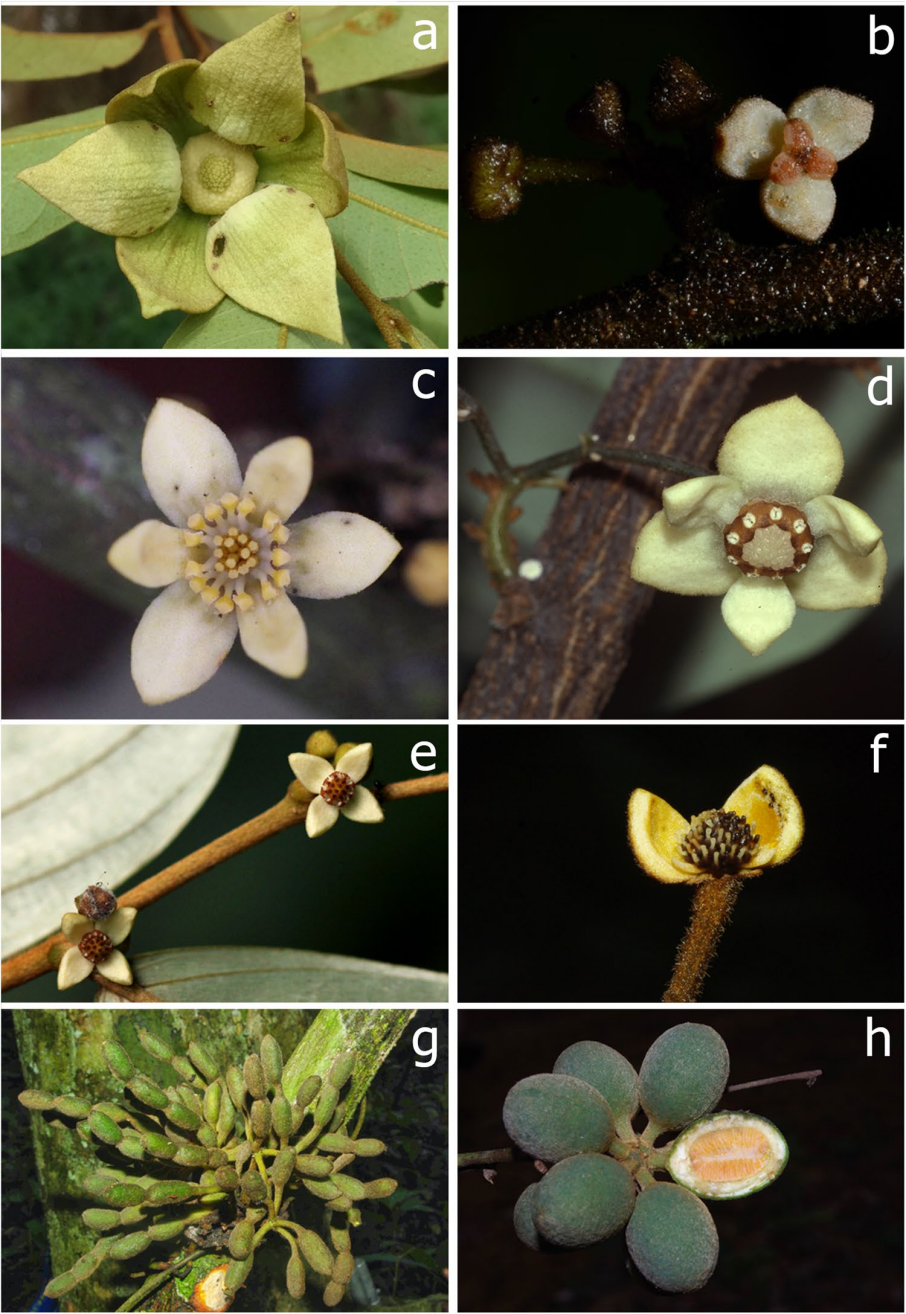
Flower morphology of outgroups (**a**) and flowers and fruits of *Monanthotaxis* (**b**–**h**). (**a**) *Uvaria scabrida*, flower showing many stamens and carpels; (**b**) *Monanthotaxis bidaultii* male flower showing three petals and three stamen; (**c**) *Monanthotaxis couvreurii*, flower showing basally fused stamens; (**d**) *Monanthotaxis whytei*, cauliflorous flower with nine hardly visible staminodes alternating with nine stamens; (**e**) flowers of *Monanthotaxis poggei* showing four petals and eight stamen, each in a single whorl; (**f**,**g**) *Monanthotaxis diclina*, female flower with one petal removed showing many carpels, and fruits showing multiple seeds per monocarp; (**h**) *Monanthotaxis paniculata* fruits with single seed per monocarp. — Photographs: (**a**) Paul H. Hoekstra, (**b**) Ehoarn Bidault; (**c**,**f**–**h**) Thomas L.P. Couvreur; (**d**) Lubbert Y.T. Westra; (**e**) Bart T. Wursten.

Several deviations from the general floral pattern in Annonaceae were observed in the genus *Monanthotaxis* during an ongoing taxonomic revision^17–19^. This genus consists of lianas and is endemic to Africa and Madagascar. With 94 species it is one of the species-rich genera of Annonaceae and the second largest genus for Africa. In contrast to most other genera in the family, *Monanthotaxis* displays a huge variation in floral characters (Fig. 1). The flowers of most species are small relative to the range of sizes observed across Annonaceae. Species such as *M. tripetala* are amongst the smallest-flowered species in the family. Petals commonly occur in either one or two whorls. In some species, however, the petals form a single whorl at the floral base, whilst the outer petals overtop the inner petals at the apex in flower buds and give the appearance of two petal whorls in bud stage. In some species (e.g. *M. bidaultii*, *M. tripetala*), reduction of floral parts had led to a decrease in size, or even absence, of the inner whorl of petals. The number of stamens greatly varies from a few stamens in a single whorl (e.g. *M. bidaultii*, *M. heterantha*), up to 120 stamens in many whorls (e.g. *M. gracilis*). Several species possess an outer whorl of staminodes^18^. In most other genera of Annonaceae these characters vary hardly.

As most species have a moderate number of small flowers high up in the canopy of tropical rainforests, almost nothing is known about the ecology and pollination biology of *Monanthotaxis*. However, well-sampled phylogenies are a helpful tool to test hypotheses about evolution^20^, facilitated by observations on floral morphology made from herbarium specimens. The exact generic delimitation of *Monanthotaxis* and allied genera had long been unclear^21^. Recently, the generic boundaries of the genus have been clarified with a well-supported phylogeny containing about 40% of the species of *Monanthotaxis*. This resulted in the transfer of the genera *Exellia, Gilbertiella* and most of the African species of *Friesodielsia* to the genus *Monanthotaxis*, rendering *Monanthotaxis* morphologically well delimited and monophyletic^19^.

In this study, we increased the taxon sampling of *Monanthotaxis* to over 75% of the species diversity. Four quantitative and two qualitative characters, which are elements of floral reductions, were scored for these species. We traced changes in these characters over the phylogeny to produce and tested hypotheses about the evolution and diversification of the genus. More specifically, we examined the evolutionary mode of the floral characters, i.e. whether these have evolved following a gradual mode or pulsed mode of evolution, and the correlations between floral characters to test the possibility that small flowers in some *Monanthotaxis* species have evolved in concert with a reduction in number of parts. This is done to create testable hypotheses about the diversification of the genus.

## Results

### Phylogenetic analyses

**Figure 2:**
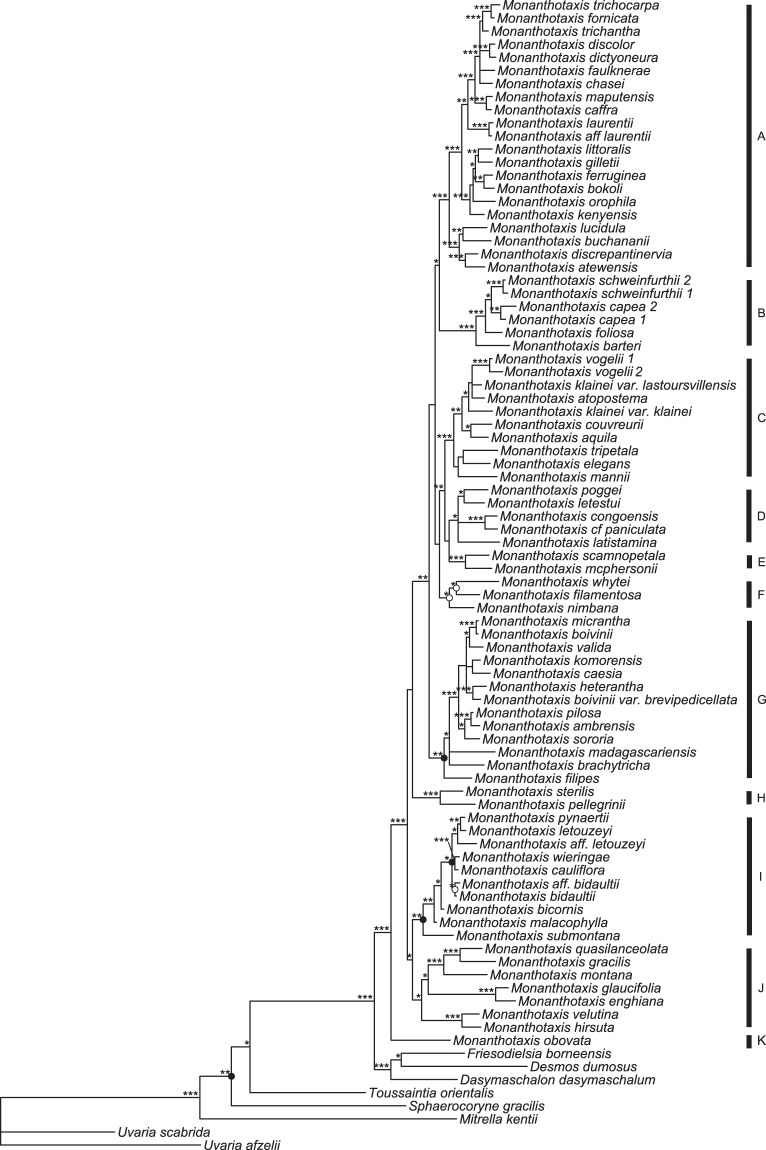
Phylogeny of *Monanthotaxis*. ***Strong node support, **moderate node support, * = weak node support. Nodes indicated with black and white dots are strongly and moderately supported, respectively, after removing species for which only a few markers were available.

The dataset used for the phylogenetic analyses comprised 6649 bp of sequence data that has been gathered for 88 specimens, including 80 specimens of *Monanthotaxis* representing 77% of the species of this genus. The best partitioning scheme found by Partitionfinder divided the nuclear and chloroplast markers in separate partitions and rendered the HKY + gamma substitution model best fitting in both cases. The analyses using CodeML did not find evidence for positive selection in the *matK* gene, while an indication for positive selection was found in 5 sites in the *rbcL* gene. Removing those five sites slightly improved the support values for a few nodes (Supplementary information Figs S1 and S2). Excluding the nine species for which less than half of the DNA sequences were available did not change the principle topology, but did improve the support values of seven nodes (Fig. 2 and Supplementary information Figs S1 and S3). As the absence of these nine species would result in the exclusion of some of the relevant morphological character variation, all subsequent analyses were done using all species, but without the five codons of *rbcL* with possible positive selection.

The genus *Monanthotaxis* was highly supported to be monophyletic in both the Bayesian and maximum likelihood analyses, with *Monanthotaxis obovata* highly supported as sister to the rest of the genus. Most backbone nodes are not well supported, in contrast to more recent nodes, notably clades A, B, C, E and H (Fig. 2 and Supplementary information Fig. S1).

Analyzing chloroplast and nuclear loci separately revealed a well-supported incongruent pattern within clade A (Supplementary information Figs S4 and S5). The nuclear data reveal a sistergroup relationship between *M. ferruginea* and *M. bokoli*, whereas the chloroplast data show that *M. ferruginea* is sister to *M. orophila* with strong support. All three species involved are closely related and differ only slightly in floral morphological characters. Therefore, we consider the effect of this incongruence on the ancestral state analyses negligible and performed all analyses after concatenation of the chloroplast and nuclear data.

### Morphological characters and analyses

**Figure 3:**
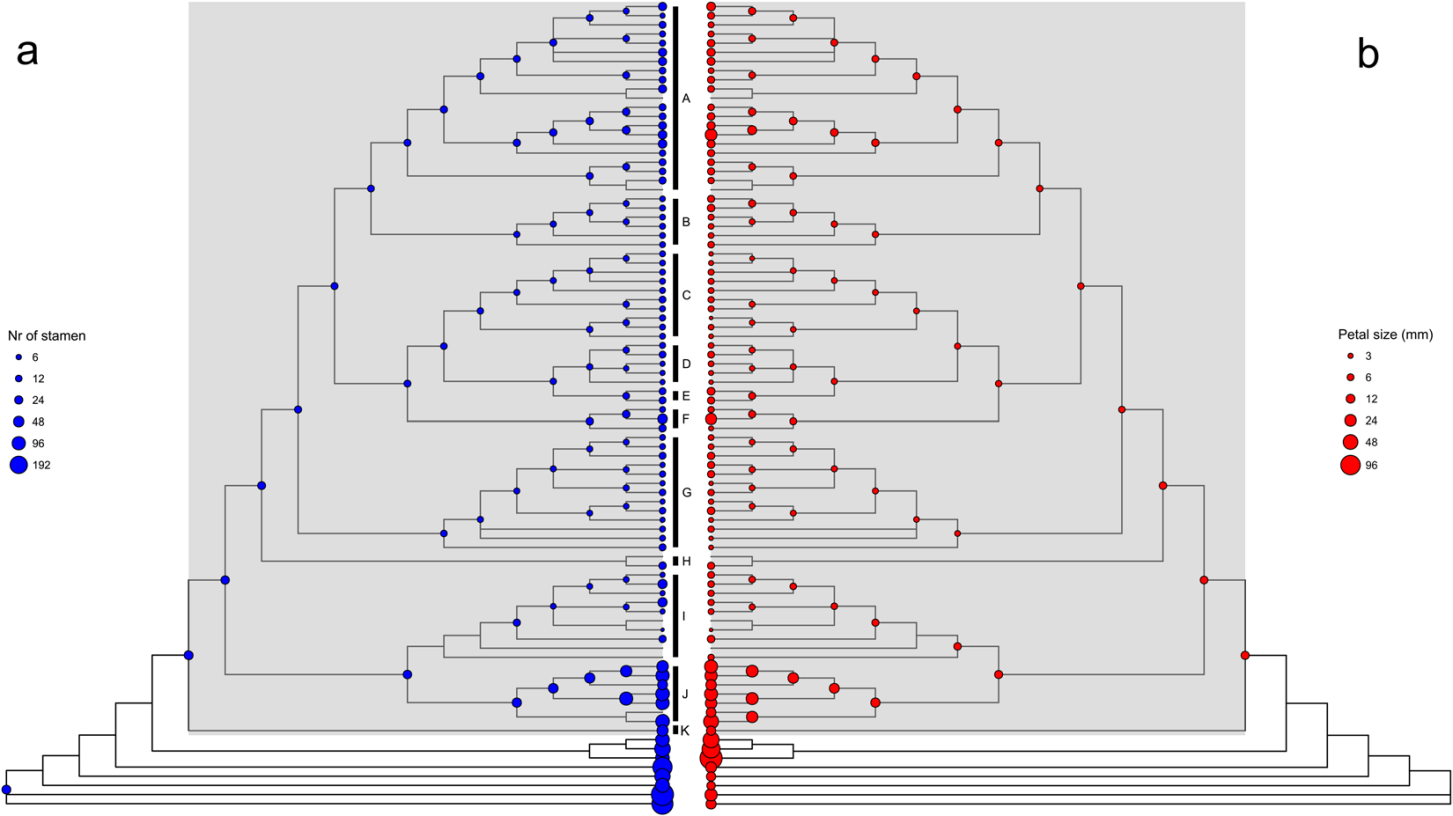
Number of stamens (**a**) and petal size in mm (**b**) plotted onto the *Monanthotaxis* phylogeny, with internal nodes showing Bayesian estimation of mean values.

**Figure 4:**
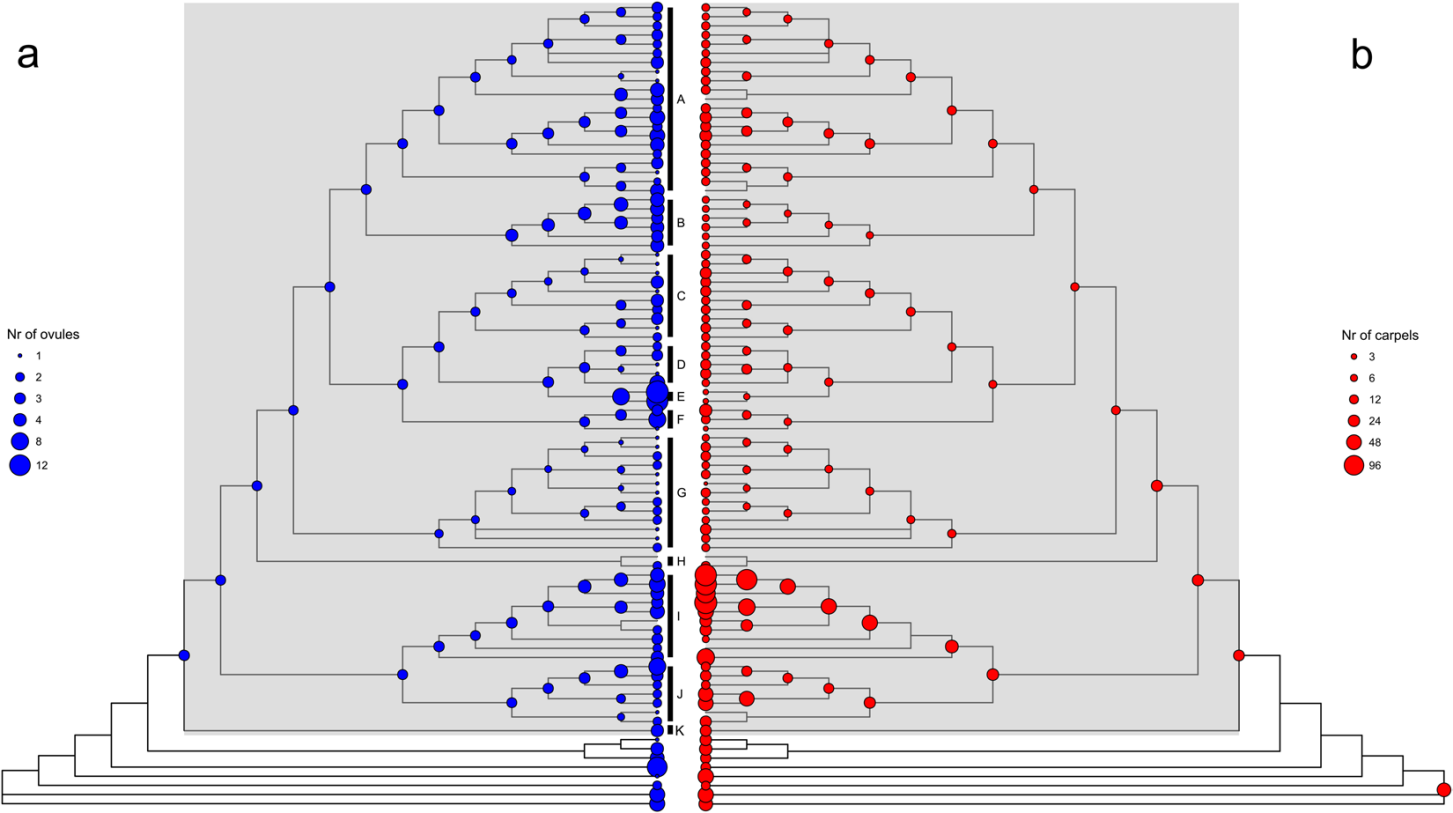
Number of ovules (**a**) and carpels (**b**) plotted on the *Monanthotaxis* phylogeny, with internal nodes showing Bayesian estimation of mean values.

**Figure 5:**
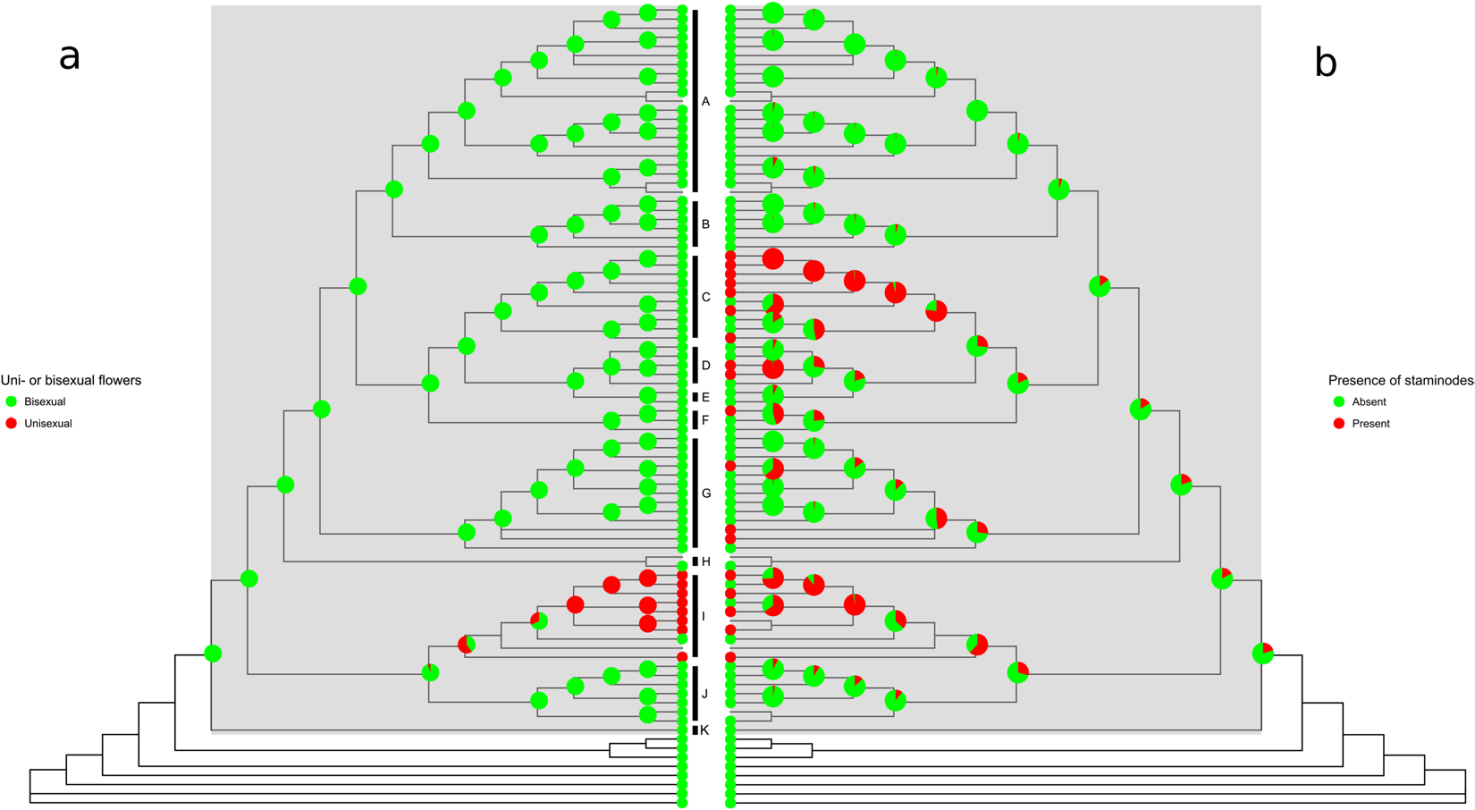
Presence of uni- or bisexual flowers (**a**) and presence of staminodes (**b**) plotted on the *Monanthotaxis* phylogeny. Pie charts show inferred states.

The results clearly show that simplification has taken place in *Monanthotaxis* with strong correlations between number of stamens, petal size and number of carpels (Table 1). Furthermore, the outgroups and clades J and K of *Monanthotaxis* have many more stamens and larger petal sizes than the remaining *Monanthotaxis* species. The number of stamens ranged from 105 to 346 in the outgroups, and from 125 in *Monanthotaxis gracilis* to as few as three in *M. bidaultii*. Maximum petal size in *Monanthotaxis* ranged from 2.2 mm in *M. tripetala* to 50 mm in *M. hirsuta*, while reaching 130 mm in the outgroup species *Dasymaschalon dasymaschalum* (Fig. 3). Due to the low support for some of the backbone nodes, it is unclear along which branch the reduction in number of stamen and petal size occurred, and whether it occurred multiple times. A general reduction in number of carpels was inferred, a trend that is reversed by an increase in carpel number in species with unisexual flowers (clade I). Some of the latter have up to 150 carpels per flower, while in some other clades the number has been reduced to a single carpel per flower. The number of ovules per carpel ranged from 1 to 16 in *Monanthotaxis* (Fig. 4). Staminodes were present in species scattered across five different clades of *Monanthotaxis* and have arisen or disappeared multiple times. Unisexual flowers were confined to a single clade (Fig. 5).

**Table 1:** Results of model tests of correlation between morphological characters.

Morphological characters	Bin/Cont	Nr of par. cor.	Nr of par. ind.	MLh cor.	MLh ind.	BF	Percentage of significant trees
Stamen number vs carpel number	Continuous	**2**	**1**	−**38.2665**	−**44.1576**	**5x V**	**76.1%**
Stamen number vs ovule number	Continuous	2	1	−34.5393	−37.3479	4x S, 1x P	55.4%
Stamen number vs petal size	Continuous	**2**	**1**	−**18.0604**	−**30.9199**	**5x V**	**100%**
Carpel number vs ovule number	Continuous	2	1	−31.3965	−32.111	1x S, 4x N	0.7%
Carpel number vs petal size	Continuous	2	1	−23.5816	−26.7138	5x S	48.4%
Ovule number vs petal size	Continuous	**2**	**1**	−**8.1434**	−**14.5965**	**4x V, 1x S**	**100%**
Staminodes vs uni/bisexual flowers	Binary	8	4	−48.167	−49.4281	5x N	0.7%

BF = results of 5 Bayes Factor tests, percentage of significant trees = percentage of trees with significant correlation using likelihood ratio test for continuous characters (p < 0.05) and using relative likelihood of AIC for the binary characters. Bin = Binary characters, cont = continuous characters. Nr of par. cor. = Number of rate parameters of model with correlation, nr of par. ind. = number of rate parameters of model without correlation. MLh cor. = Best marginal likelihood of the stepping stone sampler of the five MCMC runs of the model with correlation, MLh ind. = Best marginal likelihood of the stepping stone sampler of the five MCMC runs of the model without correlation. V = very strong indication of correlation, S = strong indication of correlation, P = positive indication of correlation, N = no or weak indication of correlation. In bold are the results which show a correlation for both types of test.

The ancestor of *Monanthotaxis* most probably had bisexual flowers and lacked staminodes (Fig. 5 and Supplementary Table S1). The ancestral state inferences of the continuous traits should be interpreted with caution, but suggest that the ancestor of *Monanthotaxis* had petals of 10 mm long, 31 stamens, 21 carpels and 3 ovules per carpel (Figs 3 and 4 and Supplementary Table S1).

Strong to very strong correlations were found between number of stamen, petal size and the number of ovules per carpel, indicating that the simplification of flowers has occurred jointly for different parts of the flowers. The number of carpels is only strongly correlated with the number of stamens when considering Bayes factors, and less strong after the likelihood ratio test (in only 76% of the input trees). The correlation between petal size and number of carpels was strong considering Bayes factors and weak between number of ovules and number of carpels, while the likelihood ratio tests found no significant correlations between these variables in the majority of input trees. The discrete tests of correlation did not retrieve evidence for a correlation between presence of staminodes and flower sexuality (Table 1).

The tests for mode of evolution significantly favoured a pulsed mode for the number of stamens and the number of carpels, indicated by kappa estimates of 0.01 and 0.1 respectively. No evidence for a significant deviation from a gradual mode of evolution was found for the number of ovules per carpel, while evidence for a deviation from a gradual mode of evolution was found using likelihood ratio tests for the petal length. The kappa estimate for petal length of 1.9 indicates that long branches contribute more to the evolution of petal length than would be expected under a gradual mode of evolution. However the Bayes factors did not indicate a deviation from a gradual mode of evolution for the petal length, suggesting that the signal is very weak if present (Table 2).

**Table 2:** Results of test for significant deviation from a Brownian motion model of evolution.

Continuous trait	MLh kappa	MLh, kappa 1	BF	Percentage of significant trees	Kappa estimate
carpels	−**18.7002**	−**20.3879**	**5x P**	**99.3%**	0.1
stamens	−**19.0699**	−**27.2545**	**5x V**	**100%**	0.01
Ovules	−12.1765	−10.7361	5x N	5.6%	0.9
petal length	−4.31857	−4.6971	5x N	92.7%	1.9

MLh kappa = Best marginal likelihood of the stepping stone sampler of the model with the estimation of kappa, MLh kappa 1 = Best marginal likelihood of the stepping stone sampler of the model with kappa set to 1. BF = Bayes factor tests, V = very strong indication, P = positive indication and N = no indication for a deviation of kappa from 1.0. In bold are the results which show a significant deviation from a Brownian motion model of evolution.

## Discussion

In this study the majority of the *Monanthotaxis* species were included in phylogenetic analyses and data of the floral characters were acquired for all species of which flowers are known. The results demonstrate that a reduction in flower size and number of floral parts has occurred in most species of *Monanthotaxis*. The methods also proved useful for the generation of new insights into the evolution of a group for which hardly any ecological information is available. In a previous study the monophyly and generic delimitation of *Monanthotaxis* were resolved^19^, based on a sample of 40% of the species of *Monanthotaxis* that still lacked some crucial species, such as those with unisexual flowers. In this study, the monophyly of *Monanthotaxis* is reaffirmed and although some nodes along the backbone of the tree receive weak support, most nodes within the genus are well supported (Fig. 2). Morphologically similar species are consistently closely related. As a result, the majority of the clades are easily recognizable by the combination of a few morphological characters. For example, the species of the well-supported clade A are distinct by the extra-axillary inflorescences, rounded flower buds, six petals in two whorls, and 30 or fewer stamens. The well-supported clade C is distinguishable by axillary inflorescences, ovate flower buds and petals in a single whorl at the base, with the outer petals overlapping the inner petals distally.

There is a trend across the phylogeny towards a reduction of stamen number and petal length in *Monanthotaxis* (Fig. 3). Indeed, all outgroup species have more than 100 stamens per flower (e.g. Fig. 1a), while the only clades of *Monanthotaxis* with more than 40 stamens per flower are the early diverging clades J and K. There are some other genera of Annonaceae which show only a few stamens, such as *Orophea*, *Bocagea* and *Miliusa*^22^. Within the tribe Uvarieae, *Monanthotaxis* is the only genus in which species with a low number of stamens occur. Consequently, the low number of stamens in most *Monanthotaxis* species is considered a reduction. Note, however, that the ancestral state for the genus *Monanthotaxis* was inferred as 31 stamen and petals of 10 mm, which is fewer than the number of stamens and a smaller petal size than the species in clades J and K, suggesting an increase has occurred in those clades. The species of these two clades were previously assigned to the genus *Friesodielsia* based on their superficial resemblance with that genus^19^.

The very strong correlation between number of stamens and petal length, and between number of stamens and number of carpels within the genus *Monanthotaxis* is consistent with the general notion that the number of floral structures depends on available space^23^. However, the reduction in flower size apparently did involve all floral parts of the species with unisexual flowers. Despite the consistent small petals, most of the unisexual flowers contain many carpels. *Monanthotaxis wieringae*, for example, has a maximum petal length of 5 mm, but has around 130 carpels. This discrepancy probably explains the weak correlation between carpel number and petal length in most of the likelihood ratio tests. However, the male flowers do follow the general reduction trend with fewer stamens in species with smaller flowers.

Pollen-ovule ratio is an indicator of the breeding system in angiosperms^24^. A high ratio indicates cross-pollination, while low pollen-ovule ratios generally are found in self-pollinating species. In view of this, the strong correlation between number of ovules per carpel and number of stamen, and between number of carpels and number of stamen, may suggest that no differences in breeding system exist within the genus *Monanthotaxis*. This is a tentative hypothesis, since the diversity in stamen forms (Fig. 1b,c,e) as well as the presence of species with unisexual flowers and bisexual flowers could indicate that different breeding systems occur in *Monanthotaxis*^25^. Moreover, it is questionable whether number of stamen can directly be linked to the number of pollen grains as there is considerable variation in anther cell size within the genus *Monanthotaxis*.

The presence of non-functional staminodes is often thought to be an intermediate stage in the reduction of stamens^10^. In the genus *Monanthotaxis* the staminodes do not have an apparent function; they form an outer whorl of reduced stamens which are often only visible as very tiny appendages less than half a millimeter in length, such as in *M. zenkeri*^18^. The fact that the presence of staminodes occurs on multiple independent branches on the phylogeny could indicate that the process of reduction is still ongoing in *Monanthotaxis*. *Monanthotaxis whytei* (Fig. 1d) is the only species on which an ontogenetic study has been performed^26^. First an outer whorl of 6 staminodes is formed; this is followed by an inner whorl of 9 staminodes then a further inner whorl of 9 stamens. The outer whorl of staminodes already stops developing early in the floral development and is not visible in the mature flowers, while the whorl of 9 staminodes is only visible as very small appendages (Fig. 1d). This could indicate that the reduction of stamens in the genus *Monanthotaxis* takes place centripetally, i.e. from the outer to the inner whorls and that the stamen whorl in species with only one whorl is homologous with the inner whorl in species with multiple whorls. It is interesting to note that the presence of staminodes in species with unisexual flowers (clade I, Fig. 1b,f,g) only occurs in male flowers and not in the female flowers a characteristic rarely reported in plants with unisexual flowers^10^.

Reduction trends can follow a gradual mode of evolution or the slightly controversial^27,28^ pulsed mode of evolution, in which rapid change is followed by periods of stasis or little changes. Genome reductions and duplications, for example, by their nature have been demonstrated to follow a pulsed mode of evolution^29,30^, but this mode of evolution has rarely been established for morphological characters. The strong indication for a pulsed mode of evolution of the number of carpels and stamens may suggest that sudden events, such as changes in environment or pollination shifts, have triggered the floral reduction in *Monanthotaxis*. It must be noted that a limitation of the test used in this study is the simultaneous inference of the mode of evolution and of the amount of morphological change associated with speciation events^28^. Currently, methods are being developed which can disentangle these questions^31^. Alternatively, the inference of a pulsed mode of evolution for the number of stamens and carpels could be explained by the reduction of entire whorls. A gradual loss of whorls will be discernable as saltational changes in numbers of individual stamens and carpels, given the high number of floral parts per whorl. The irregular pattern of stamens and carpels in some species hampered the observation of the number of whorls in this study. Ontogenetic studies are needed for *Monanthotaxis* species with higher numbers of stamens and carpels to assess the exact number of whorls and assess the mode of evolution of those characteristics.

In general, we demonstrate an evolutionary reduction of flowers with a high number of floral parts to reduced flowers with as few as three petals and three stamens in the genus *Monanthotaxis* (Fig. 1b). The question remains whether a shift in pollination regime has triggered this reduction. In most species of Annonaceae the flowers are pollinated by beetles. Self-pollination is generally prevented by protogyny, viz. flowers entering the pistillate phase first, with a subsequent non-sexual phase and finally the staminate phase^32^. This is also the case in the genera *Desmos*, *Dasymaschalon* and *Friesodielsia*, the genera most closely related with *Monanthotaxis* for which pollination studies exist^32^. Protogyny also occurs in *Monanthotaxis* as in herbarium specimens only flowers in the pistillate phase or staminate phase have been observed. Flowers with overlapping staminate and pistillate functional phases have not been observed. The pollinators of *Monanthotaxis* however are unknown. Despite great differences in petal morphology in the tribe Uvarieae, most species are reported to be pollinated by beetles. However, pollination by cockroaches as well as bees has been reported in the genus *Uvaria* and flies and thrips have been reported as pollinators in some other genera outside the Uvarieae^32^. Small unisexual flowers, as those of most *Monanthotaxis* species in clade I, have been correlated with fly as well as wind pollination^25^. However, without further studies the question remains if the great diversity in stamen forms and number in *Monanthotaxis* also reflect a diversity in pollination syndromes in this genus.

*Monanthotaxis* is the second-most diverse genus of Annonaceae in Africa, especially in central Africa as many as 10 species can occur sympatrically. The majority of these sympatric species belong to different subclades, with most pairs of sister species showing allopatric distributions. Therefore, the diversification of *Monanthotaxis* may have been promoted by floral adaptations of different subclades. This enabled them to occupy different ecological microniches and consequently species of different clades are able to co-occur. Dispersal limitation could have promoted subsequent diversification. The pattern of allopatric and sympatric distributions can only be tested directly if more accurate distribution data become available. Recent expeditions have shown that the known distributions of species are larger than previously thought and these also uncovered many undescribed species^18^ showing that the genus is undersampled in some areas of Africa. Finally, follow-up studies on the ontogeny, pollination and ecology are needed to understand more about the diversification of the genus *Monanthotaxis* and to answer questions on biodiversity of tropical rain forests.

## Methods

### Taxon sampling

For each species, subspecies and variety of *Monanthotaxis* at least one representative voucher specimen was selected and six species have been included twice in this study as these belong to allopatric populations with some morphological differences. Eight outgroup species were selected based on the phylogeny of Guo *et al*.^19^ including all genera in the *Dasymaschalon* clade and representative genera of the tribe Uvarieae. 262 DNA sequences were newly generated for this study and 299 sequences were taken from previous studies^19,33^. All voucher specimens were identified by the first author in the framework of a taxonomic revision of the genus *Monanthotaxis*. Sequences for a total of 72 out of the 94 species (74 of 96 taxa when including varieties) of *Monanthotaxis* were successfully produced. This includes nine recently described new species^17,18^ and six undescribed species (Hoekstra unpublished). An initial exploration of specimens from Madagascar indicated that there are at least 12 undescribed species from that region. Of the 22 taxa not included 8 species were only very recently recognized as new species to science. The other taxa are only known from very old collections and DNA extraction and amplification failed or no permission was obtained from the herbaria to sample those old collections.

### DNA extraction, amplification and sequencing

DNA sequences of five chloroplast markers (*matK*, *ndhF*, *psbA-trnH*, *rbcL* and *trnL-F*) and two nuclear markers (ETS and ITS1-5.8S-ITS2) were generated for 42 specimens and downloaded from genbank for 46 specimens.

DNA extraction, amplification and sequencing followed the protocol that has been previously described^34,35^ and the PCR reaction for the nuclear markers as in Guo *et al*.^19^ with the modification that 5 µl of 5x Betaine was added to each 25 µl PCR, because the ITS and ETS sequences have a high GC content (55–60%). The PCR products were sequenced on an ABI 3730 Sequencing platform by BaseClear (Leiden, The Netherlands). For voucher information and GenBank accession numbers see Supplementary Table S2.

### Phylogenetic analyses

Trace files were checked and assembled in Sequencer 5.4 (Gene Codes Corporation, MI U.S.A.) or BioEdit 7.2.5^36^. Sequences were aligned using the L-ins-I option in MAFFT v 7.245^37^ and verified manually in Mesquite v 3.11^38^. Ambiguously aligned regions (in ITS and ETS) as well as microsatellites were excluded from the analyses^33^. Some species had an inversion of 14 positions in the *psbA*-*trnH* spacer. Following Pirie *et al*.^39^, this fragment was inverted when necessary, to retain any phylogenetic information. Gaps were coded following the method of Simmons and Ochoterena^40^ using FastGap 1.2^41^.

Phylogenetic trees were inferred using maximum parsimony analyses, maximum likelihood analyses and Bayesian Inference. With the program Partitionfinder v. 1.1.1^42^ we inferred the best partitioning scheme as well as the best substitution models for those partitions. Individual loci, and separate codon positions for the coding genes, were defined as the data blocks. All possible partitioning schemes and substitution models were fitted on the dataset using the greedy algorithm of PhyML in Partitionfinder and the best partitioning scheme was selected based on the Bayesian Information Criterion.

Maximum parsimony analyses were performed in PAUP* version 4.0a151^43^ with the heuristic search option and tree bisection-reconnection (TBR) branch swapping, with 1000 random addition sequence replicates, saving 50 trees per replicate. Character states were treated as unordered and of equal weight^44^. To assess clade support nonparametric bootstrap analyses were performed with 1000 bootstrap replicates and 100 random addition sequence replicates per bootstrap replicate, saving 50 trees per replicate.

Bayesian inference was conducted with MrBayes v3.2.6^45^ on the CIPRES gateway server^46^. The best fitting model of sequence evolution as defined by Partitionfinder was applied to the DNA sequence data and the restriction site model with the setting “coding = variable” was applied to the gap-coding data. It has been shown that MCMC analyses of closely related species may get biased towards excessively long branch-length estimates^47^ and therefore following Guo *et al*.^19^, the temperature parameter was set to 0.08 and the mean branch length prior was set to 0.01 (brlenspr = unconstrained: exponential (100.0)). These settings improved mixing of the chains compared to the default values of the program. Four different chains were run for 10 million generations sampling every 1000^th^ generation. Convergence was assessed using the R-package RWTY version 1.0.1^48^ as this package includes an estimation of the effective sample size (ESS) for the tree topology parameter^49^ as well as ESS values for other parameters and diagnostic plots.

Maximum likelihood analyses were run on the CIPRES Gateway server with RAxML version 8^50^. The GTR-gamma model of substitutions was used for the DNA sequence data and the bingamma model for the gap-coded data. To assess clade support a rapid bootstrap analysis was performed on the best-scoring tree with 1000 nonparametric bootstrap iterations.

In many angiosperms positive selection takes place on the active sites of the *rbcL*-gene and removing those sites could improve phylogenetic resolution^51^. This has been found in a family-wide study of Annonaceae^52^. We tested for signs of positive selection in the genes *rbcL* and *matK*, using the branch-site model of positive selection^53^ in the program Codeml of the PAML4.8 package^54^. A simplified version without branch lengths of the most likely tree from the maximum likelihood analyses was used as a backbone phylogeny. We assessed the presence of positive selection for the branch subtending the *Monanthotaxis* crown node, for each gene separately. These models were compared using a likelihood ratio test against a null model in which the value ω was fixed. When the likelihood ratio test significantly demonstrated the presence of positive selection (P < 0.05), the Bayes empirical Bayes (BEB)^53^ was used to calculate the posterior probabilities for site classes and to identify the sites under positive selection. These codons were deleted from the alignment and subsequently all phylogenetic inferences were redone with the new dataset without codons under positive selection.

Bayesian, maximum likelihood and maximum parsimony analyses were rerun separately on the nuclear and chloroplast loci to check for incongruences. Nine specimens, for which more than half of the DNA sequences were missing (3000 bp), were excluded from the dataset and all phylogenetic inferences were rerun to test if support values for any nodes improved. The support values from all different runs were summarized and compared using a customized Python script with the library Dendropy version 4.1.0^55^. Maximum parsimony and maximum likelihood bootstrap values from 85 to 100 were considered as strong node support, values from 75 to 84 as moderate support and values from 50 to 74 as weak support. Posterior probabilities higher than 0.95 were considered as supported, below 0.95 as unsupported.

### Morphological character sampling

Six morphological characters were selected to be used as a proxy for floral reduction. Four characters are quantitative, i.e. maximum petal length, number of stamens, number of carpels and number of ovules per carpel, and two characters are qualitative, i.e. flower sexuality and presence of staminodes. For species of *Monanthotaxis* all these characters were (re-)measured from herbarium material as in published literature sometimes errors were encountered. For the outgroup species data was taken from literature^56–58^ and missing data (including number of stamen for all species) was measured from herbarium material.

Quantitative variables were transformed to logarithmic scale. However, variation occurs in the number of stamens and carpels within species and even within specimens, with variation increasing with the number of stamens and carpels per species. Species with generally nine stamens often may have a few flowers with eight stamens and sometimes even twelve stamens, while in a species with around 100 stamens the number can vary from 80 to 120 stamens. To take the increasing absolute value of variation with increasing number of stamens and or carpels into account, the average of the logarithm of the minimum and maximum number of stamens and carpels per species was used in the analyses.

The logarithm of the average of the maximum and minimum number of ovules per carpel was taken for species with a variable number of ovules per carpels. Species with a single ovule per carpel rarely have carpels with two ovules, and vice versa; these species were scored as having the predominantly occurring number of ovules.

The morphological characters could be observed for almost all species, with the exception of *Monanthotaxis sterilis* for which neither flowers nor fruits are known. Other species for which some of the characters could not be scored were *M. atewensis, M. malacophylla* and *M*. aff. *laurentii* for which only fruits are known, *M. aff. bidaultii* and *M. submontana* for which only female flowers are available, and *M. velutina*, the only flowering specimen has old flowers.

### Character mapping and analyses

Morphological characters were optimized and analyzed on phylogenetic trees using BayesTraits version 2.0^59^. One thousand randomly chosen trees from the Bayesian analyses, after discarding the burnin and outgroup species, were selected to account for phylogenetic uncertainty.

Throughout the analyses in BayesTraits, model testing was used to test whether more complex models better fitted the data than simpler models. Three different model tests were performed: likelihood ratio tests were used for Maximum Likelihood (ML) analyses for nested models, AIC relative likelihoods were used for Maximum Likelihood (ML) analyses of non-nested models and Bayes factors for Markov Chain Monte Carlo (MCMC) methods. The likelihood ratio tests were performed for each of the one thousand input trees based on the complex and simpler models and for each tree was tested if the p-value of the likelihood ratio statistic was below 0.05. For the AIC-test, the AIC values were calculated for each of the one thousand input trees of the complex and simpler model. Next, the relative likelihood of the simpler model was calculated and values less than 0.05 were considered evidence that the complex model better fitted the data than the simpler model. For the MCMC analyses Bayes factors were calculated from the marginal likelihoods using a stepping stone sampler^60^, as marginal likelihoods estimated by a stepping stone sampler have been shown to be more robust than the harmonic mean marginal likelihoods^61^. One hundred stones were used to calculate an estimate of the marginal likelihood and each stone was run for 10,000 iterations. Bayes factors higher than 2 were considered positive evidence, higher than 5 strong evidence and higher than 10 as very strong evidence for selection of the more complex model compared to the simpler model with less parameters.

To infer the ancestral states of the discrete characters, a continuous-time Markov model using MCMC methods^62^ was applied. Hyper-priors were used to seed the mean of the exponential priors from a uniform distribution ranging from 0 to 100. Model tests using Bayes factors were applied to test whether different transition rates from one state to another state better fitted the data than both rates set equal. For the character of uni- or bisexual flowers, the Bayes factors did not reject the hypothesis that the transition rates are equal, this was accommodated in the prior settings. While equal rates were significantly rejected (P < 0.05) for the ancestral states of the staminodes presence and those were inferred using reversible-jump Markov Chain Monte Carlo^63^.

A two-step process was run to infer the ancestral states of the continuous characters. First an MCMC with a continuous random walk model^64^ was run to estimate a distribution of models from the available data and subsequently these models were used to infer the ancestral states. Bayes factors of initial analyses indicated that the trait ‘number of stamens’ does not evolve along the phylogeny following a Brownian motion model of evolution. Therefore, the lambda parameter was set to be estimated in analyses with the number of stamens character. Finally, after discarding the burnin, the mean and standard deviation of the ancestral state inferences were calculated for each node.

For both the discrete and continuous characters five runs using MCMC methods were performed for each trait to infer the ancestral nodes. The runs consisted of 10 million generations with a burnin of 100,000 generations and sampling every 10,000 generations. Convergence was checked by calculating the ESS-values with the R-package coda version 0.19–1 and by verifying if the acceptance rate for proposed changes to the chain lies between 20 and 40%.

To test for correlated evolution between 2 character traits, both 5 MCMC and 2 ML analyses were run for each model. The binary traits were first run with the discrete independent model in BayesTraits in which the traits are assumed to have evolved independently. Then the traits were run with the discrete dependent model which assumes that the traits are correlated. For the continuous characters they were first run with the continuous random walk model and subsequently those analyses were rerun, but with the correlation between the two traits being forced to be zero. Both Bayes Factors and likelihood ratio tests were performed to test if the models that assume correlation between the traits better fitted the data than the models without such correlation.

We tested whether the four continuous morphological characters evolved along the phylogeny following a pulsed or a gradual mode of trait evolution. In the same analyses we also tested if morphological changes concentrate around speciation events. First, analyses were run with the kappa scaling parameter set to be estimated and subsequently run with the kappa parameter fixed to 0 and additionally run with the kappa parameter fixed to 1. A kappa close to 0 indicates a pulsed mode of evolution for the involved trait, while a kappa of 1 indicates a gradual mode of evolution. Values lower than 1 indicate that shorter branches contribute more to the character evolution, while values higher than 1 indicate that longer branches contribute more. Model testing as described for the correlation tests were used to test if the kappa parameter significantly differed from 1 and/or from 0.

## Electronic supplementary material

Figures S1 to S5

Table S1 to S3

## Data Availability

All sequences are available in genbank, for voucher information and genbank accession numbers see Supplementary Table S2. The morphological character matrix used in the analyses is available in Supplementary Table S3.

## References

[1] O’Malley MA, Wideman JG, Ruiz-Trillo I (2016). Losing complexity: the role of simplification in macroevolution. Trends Ecol. Evol..

[2] Wicke S (2013). Mechanisms of functional and physical genome reduction in photosynthetic and nonphotosynthetic parasitic plants of the broomrape family. Plant Cell.

[3] Holland PWH (2013). Evolution of homeobox genes. Wiley Interdiscip. Rev. Dev. Biol..

[4] Graham SW, Lam VKY, Merckx VSFT (2017). Plastomes on the edge: the evolutionary breakdown of mycoheterotroph plastid genomes. New Phytol..

[5] Sauquet H (2017). The ancestral flower of angiosperms and its early diversification. Nat. Commun..

[6] Mitchell CH, Diggle PK (2005). The evolution of unisexual flowers: morphological and functional convergence results from diverse developmental transitions. Am. J. Bot..

[7] Friedman J, Barrett SCH (2008). A phylogenetic analysis of the evolution of wind pollination in the angiosperms. Int. J. Plant Sci..

[8] Goodwillie C, Ness JM (2005). Correlated evolution in floral morphology and the timing of self-compatibility in *Leptosiphon jepsonii* (Polemoniaceae). Int. J. Plant Sci..

[9] Goodwillie C (2010). Correlated evolution of mating system and floral display traits in flowering plants and its implications for the distribution of mating system variation. New Phytol..

[10] Walker-Larsen J, Harder LD (2000). The evolution of staminodes in angiosperms: patterns of stamen reduction, loss, and functional re-invention. Am. J. Bot..

[11] Arber EAN, Parkin J (1907). On the origin of angiosperms. J. Linn. Soc., Bot..

[12] Friis EM, Pedersen KR, Crane PR (2006). Cretaceous angiosperm flowers: Innovation and evolution in plant reproduction. Palaeogeogr. Palaeoclimatol. Palaeoecol..

[13] Endress PK, Doyle JA (2009). Reconstructing the ancestral angiosperm flower and its initial specializations. Am. J. Bot..

[14] Rainer, H. & Chatrou, L. W. *AnnonBase: world species list of Annonacea*e, http://www.sp2000. org and http://www.annonaceae.org (2006). Access date: 17 April 2018.

[15] Cardoso D (2017). Amazon plant diversity revealed by a taxonomically verified species list. Proceedings of the National Academy of Sciences.

[16] Endress PK, Armstrong JE (2011). Floral development and floral phyllotaxis in *Anaxagorea* (Annonaceae). Ann. Bot..

[17] Hoekstra PH, Chatrou LW, Wieringa JJ (2014). A new species of *Monanthotaxis* from Gabon with a unique inflorescence type for Annonaceae. Phytotaxa.

[18] Hoekstra, P. H., Wieringa, J. J. & Chatrou, L. W. A nonet of novel species of *Monanthotaxis* (Annonaceae) from around Africa. *PhytoKeys*, 71–103, 10.3897/phytokeys.69.9292 (2016). 10.3897/phytokeys.69.9292 PMC5029142 27698586

[19] Guo X (2017). Cutting up the climbers: Evidence for extensive polyphyly in *Friesodielsia* (Annonaceae) necessitates generic realignment across the tribe Uvarieae. Taxon.

[20] Soltis PS, Soltis DE, Chase MW (1999). Angiosperm phylogeny inferred from multiple genes as a tool for comparative biology. Nature.

[21] Wang J (2012). A plastid DNA phylogeny of *Dasymaschalon* (Annonaceae) and allied genera: Evidence for generic non-monophyly and the parallel evolutionary loss of inner petals. Taxon.

[22] Van Heusden E (1992). Flowers of annonaceae. Blumea Suppl..

[23] Endress PKS (1994). sizes and evolutionary trends in stamens of Magnoliidae. Bot. Jahrb. Syst..

[24] Cruden RW (1977). Pollen-ovule ratios: A conservative indicator of breeding systems in flowering plants. Evolution.

[25] Charlesworth D (1993). Why are unisexual flowers associated with wind pollination and unspecialized pollinators?. Am. Nat..

[26] Ronse Decraene LP, Smets E (1990). The floral development of Popowia whitei (Annonaceae). Nord. J. Bot..

[27] Theißen G (2009). Saltational evolution: hopeful monsters are here to stay. Theory in Biosciences.

[28] Pennell MW, Harmon LJ, Uyeda JC (2014). Is there room for punctuated equilibrium in macroevolution?. Trends Ecol. Evol..

[29] Adams KL, Qiu Y-L, Stoutemyer M, Palmer JD (2002). Punctuated evolution of mitochondrial gene content: High and variable rates of mitochondrial gene loss and transfer to the nucleus during angiosperm evolution. Proceedings of the National Academy of Sciences.

[30] Wolf YI, Koonin EV (2013). Genome reduction as the dominant mode of evolution. BioEssays.

[31] Landis MJ, Schraiber JG (2017). Pulsed evolution shaped modern vertebrate body sizes. Proceedings of the National Academy of Sciences.

[32] Saunders RMK (2012). The diversity and evolution of pollination systems in Annonaceae. Bot. J. Linn. Soc..

[33] Chatrou LW (2012). A new subfamilial and tribal classification of the pantropical flowering plant family Annonaceae informed by molecular phylogenetics. Bot. J. Linn. Soc..

[34] Guo X (2014). Reassessing the taxonomic status of two enigmatic Desmos species (Annonaceae): Morphological and molecular phylogenetic support for a new genus, Wangia. Journal of Systematics and Evolution.

[35] Thomas DC (2012). Molecular phylogenetics and historical biogeography of the *Meiogyne*-*Fitzalania* clade (Annonaceae): Generic paraphyly and late Miocene-Pliocene diversification in Australasia and the Pacific. Taxon.

[36] Hall TA (1999). BioEdit: a user-friendly biological sequence alignment editor and analysis program for Windows 95/98/NT. Nucleic Acids Symp. Ser..

[37] Katoh K, Standley DM (2013). MAFFT multiple sequence alignment software version 7: improvements in performance and usability. Mol. Biol. Evol..

[38] Maddison, W. P. & Maddison, D. R. *Mesquite: a modular system for evolutionary analysis. Version 3.11*, http://mesquiteproject.org Access date (2016).

[39] Pirie MD, Chatrou LW, Mols JB, Erkens RHJ, Oosterhof J (2006). ‘Andean-centred’ genera in the short-branch clade of Annonaceae: testing biogeographical hypotheses using phylogeny reconstruction and molecular dating. J. Biogeogr..

[40] Simmons MP, Ochoterena H (2000). Gaps as characters in sequence-based phylogenetic analyses. Syst. Biol..

[41] Borchsenius, F. *FastGap 1.2*, http://www.aubot.dk/FastGap_home.htm Access date (2009).

[42] Lanfear R, Calcott B, Ho SYW, Guindon S (2012). PartitionFinder: Combined selection of partitioning schemes and substitution models for phylogenetic analyses. Mol. Biol. Evol..

[43] Swofford, D. L. *PAUP*. Phylogenetic analysis using parsimony (* and other methods), version 4*, http://paup.sc.fsu.edu/ (2002). Access date: 11 November 2016.

[44] Fitch WM (1971). Toward defining the course of evolution: minimum change for a specific tree topology. Syst. Biol..

[45] Ronquist F, Huelsenbeck JP (2003). MrBayes 3: Bayesian phylogenetic inference under mixed models. Bioinformatics.

[46] Miller, M. A., Pfeiffer, W. & Schwartz, T. in *Gateway Computing Environments Workshop (GCE)*. 1–8 (IEEE, 2010).

[47] Brown JM, Hedtke SM, Lemmon AR, Lemmon EM (2010). When trees grow too long: Investigating the causes of highly inaccurate bayesian branch-length estimates. Syst. Biol..

[48] Warren, D., Geneva, A., Swofford, D. & Lanfear, R. *rwty: R We There Yet*, https://CRAN.R-project.org/package=rwty Access date (2016).

[49] Lanfear R, Hua X, Warren DL (2016). Estimating the effective sample size of tree topologies from bayesian phylogenetic analyses. Genome Biol. Evol..

[50] Stamatakis A (2014). RAxML version 8: a tool for phylogenetic analysis and post-analysis of large phylogenies. Bioinformatics.

[51] Kapralov, M. V. & Filatov, D. A. Widespread positive selection in the photosynthetic Rubisco enzyme. *BMC Evol. Biol*. **7**, 10.1186/1471-2148-7-73 (2007). 10.1186/1471-2148-7-73 PMC1884142 17498284

[52] Hoekstra PH (2017). Correlated evolutionary rates across genomic compartments in Annonaceae. Mol. Phylogenet. Evol..

[53] Yang Z, Wong WSW, Nielsen R (2005). Bayes empirical bayes inference of amino acid sites under positive selection. Mol. Biol. Evol..

[54] Yang Z (2007). PAML 4: Phylogenetic analysis by maximum likelihood. Mol. Biol. Evol..

[55] Sukumaran J, Holder MT (2010). DendroPy: A Python library for phylogenetic computing. Bioinformatics.

[56] Verdcourt, B. In *Flora of tropical East Africa* Vol. 70 (eds Milne-Redhead, E. & Polhill, R.M.) 1–132 (East African Community by the Crown Agents for Oversea Governments and Administrations, 1971).

[57] Le Thomas, A. In *Flore du Gabon Vol. 16* (ed Aubréville, A.) 1–371 (Muséum National d’Histoire Naturelle, 1969).

[58] Turner I (2012). Annonaceae of Borneo: A review of the climbing species. Gard. Bull. Singapore.

[59] Pagel, M. & Meade, A. *BayesTraits V2*.0, http://www.evolution.rdg.ac.uk/BayesTraits.html Access date (2014).

[60] Xie W, Lewis PO, Fan Y, Kuo L, Chen M-H (2011). Improving marginal likelihood estimation for bayesian phylogenetic model selection. Syst. Biol..

[61] Baele G (2012). Improving the accuracy of demographic and molecular clock model comparison while accommodating phylogenetic uncertainty. Mol. Biol. Evol..

[62] Pagel M, Meade A, Barker D, Thorne J (2004). Bayesian estimation of ancestral character states on phylogenies. Syst. Biol..

[63] Pagel M, Meade A (2006). Bayesian analysis of correlated evolution of discrete characters by reversible-jump markov chain monte carlo. Am. Nat..

[64] Pagel, M. In *Morphology, shape and phylogeny Systematics Association Special Volume* Series (eds Norman MacLeod & Peter L Forey) Ch. 13, 269–286 (CRC Press, 2002).

