# Supplementary material for: Floral evolution by simplification in Monanthotaxis (Annonaceae) and hypotheses for pollination system shifts
Source: Sci Rep. 2018 Aug 13;8:12066. doi: 10.1038/s41598-018-30607-2 (PMC6089970; doi:10.1038/s41598-018-30607-2)
Supplement: Supplementary file 1 — Figures S1 to S5 [file 41598_2018_30607_MOESM1_ESM.pdf]

Scientific reports Supplementary Information Figures S1 to S5

" Floral evolution by simplification in Monanthotaxis (Annonaceae) and hypotheses for pollination system shifts."

Paul H. Hoekstra*, Jan J. Wieringa, Erik Smets, Lars W. Chatrou

*corresponding author, paul.hoekstra@naturalis.nl

Supplementary information Figure S1. Phylogeny of *Monanthotaxis* as used for the analyses. Node support values indicate respectively maximum parsimony bootstrap support, Maximum likelihood bootstrap support and Bayesian posterior probabilities. Hyphens denote bootstrap values < 50.

Supplementary information Figure S2. Phylogeny of *Monanthotaxis*. Result of analyses with all sequences, including the five codons of RBCL for which positive selection was found. Node support values indicate respectively maximum parsimony bootstrap support, Maximum likelihood bootstrap support and Bayesian posterior probabilities. Hyphens denote bootstrap values < 50.

Supplementary information Figure S3. Phylogeny of *Monanthotaxis* without nine specimens for which less than half of the sequences was available. Node support values indicate respectively maximum parsimony bootstrap support, Maximum likelihood bootstrap support and Bayesian posterior probabilities. Hyphens denote bootstrap values < 50.

Supplementary information Figure S4. Phylogeny of *Monanthotaxis* based on only the chloroplast sequences. Node support values indicate respectively maximum parsimony bootstrap support, Maximum likelihood bootstrap support and Bayesian posterior probabilities. Hyphens denote bootstrap values < 50.

Supplementary information Figure S5. Phylogeny of *Monanthotaxis* based on only the nuclear sequences. Node support values indicate respectively maximum parsimony bootstrap support, Maximum likelihood bootstrap support and Bayesian posterior probabilities. Hyphens denote bootstrap values < 50.

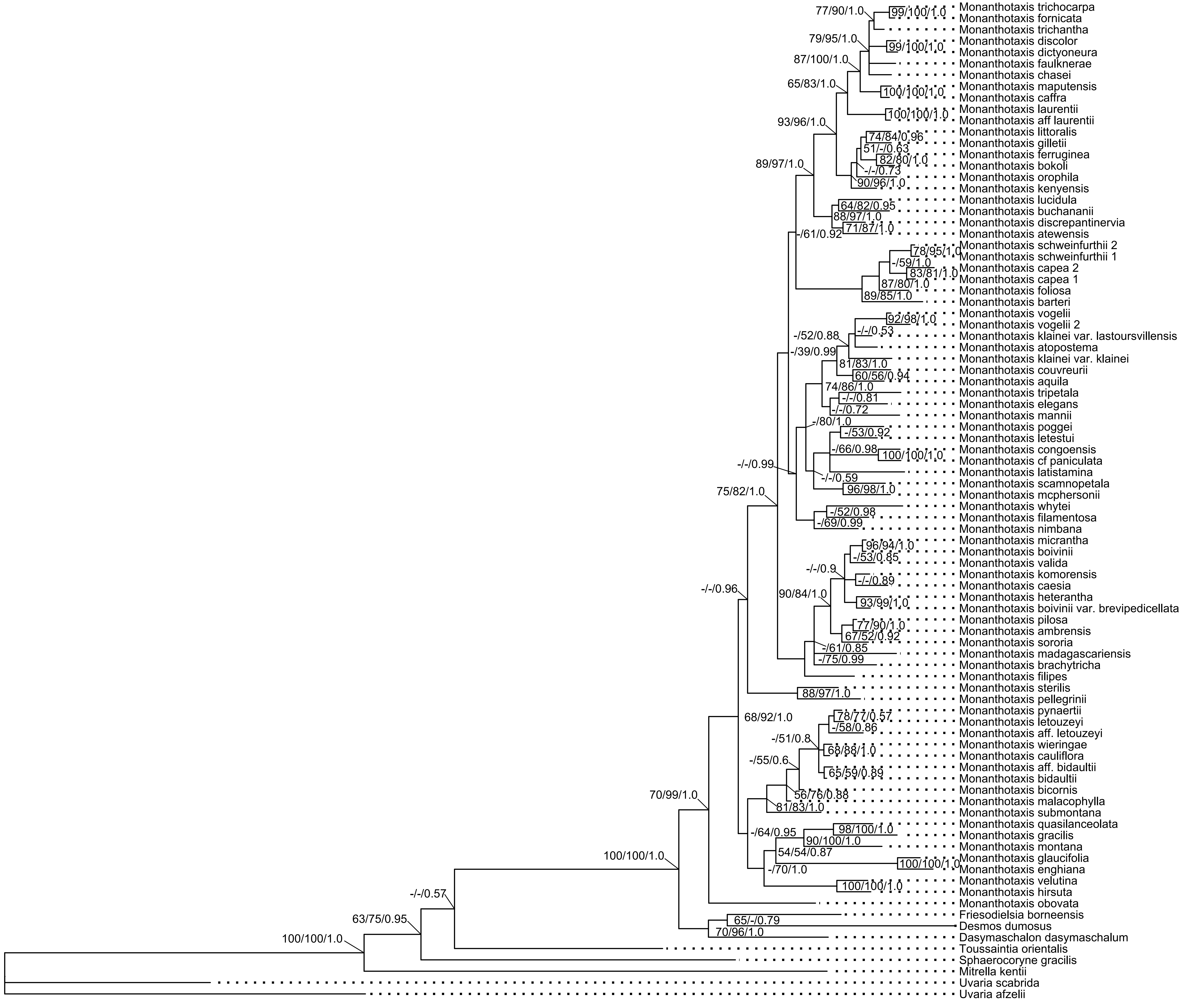
77/90/1.0
99/100/1.0
79/95/1.0
99/100/1.0
87/100/1.0
65/83/1.0
100/100/1.0
100/100/1.0
93/96/1.0
74/84/0.96
51/-/0.63
82/80/1.0
-/-/0.73
90/96/1.0
89/97/1.0
64/82/0.95
88/97/1.0
71/87/1.0
-/61/0.92
78/95/1.0
-/59/1.0
83/81/1.0
87/80/1.0
89/85/1.0
92/98/1.0
-/-/0.53
-/52/0.88
-/39/0.99
81/83/1.0
60/56/0.94
74/86/1.0
-/-/0.81
-/-/0.72
-/80/1.0
-/53/0.92
-/66/0.98
100/100/1.0
-/-/0.99
-/-/0.59
96/98/1.0
75/82/1.0
-/52/0.98
-/69/0.99
96/94/1.0
-/53/0.85
-/-/0.9
-/-/0.89
90/84/1.0
93/99/1.0
77/90/1.0
67/52/0.92
-/61/0.85
-/75/0.99
-/-/0.96
88/97/1.0
68/92/1.0
78/77/0.57
-/58/0.86
-/51/0.8
68/88/1.0
-/55/0.6
65/59/0.89
56/76/0.88
70/99/1.0
81/83/1.0
98/100/1.0
-/64/0.95
90/100/1.0
54/54/0.87
100/100/1.0
-/70/1.0
100/100/1.0
100/100/1.0
-/-/0.57
65/-/0.79
63/75/0.95
70/96/1.0
100/100/1.0
Monanthotaxis trichocarpa
Monanthotaxis fornicata
Monanthotaxis trichantha
Monanthotaxis discolor
Monanthotaxis dictyoneura
Monanthotaxis faulknerae
Monanthotaxis chasei
Monanthotaxis maputensis
Monanthotaxis caffra
Monanthotaxis laurentii
Monanthotaxis aff laurentii
Monanthotaxis littoralis
Monanthotaxis gilletii
Monanthotaxis ferruginea
Monanthotaxis bokoli
Monanthotaxis orophila
Monanthotaxis kenyensis
Monanthotaxis lucidula
Monanthotaxis buchananii
Monanthotaxis discrepantinervia
Monanthotaxis atewensis
Monanthotaxis schweinfurthii 2
Monanthotaxis schweinfurthii 1
Monanthotaxis capea 2
Monanthotaxis capea 1
Monanthotaxis foliosa
Monanthotaxis barteri
Monanthotaxis vogelii
Monanthotaxis vogelii 2
Monanthotaxis klainei var. lastoursvillensis
Monanthotaxis atopostema
Monanthotaxis klainei var. klainei
Monanthotaxis couvreurii
Monanthotaxis aquila
Monanthotaxis tripetala
Monanthotaxis elegans
Monanthotaxis mannii
Monanthotaxis poggei
Monanthotaxis letestui
Monanthotaxis congoensis
Monanthotaxis cf paniculata
Monanthotaxis latistamina
Monanthotaxis scamnopetala
Monanthotaxis mcphersonii
Monanthotaxis whytei
Monanthotaxis filamentosa
Monanthotaxis nimbana
Monanthotaxis micrantha
Monanthotaxis boivinii
Monanthotaxis valida
Monanthotaxis komorensis
Monanthotaxis caesia
Monanthotaxis heterantha
Monanthotaxis boivinii var. brevipedicellata
Monanthotaxis pilosa
Monanthotaxis ambrensis
Monanthotaxis sororia
Monanthotaxis madagascariensis
Monanthotaxis brachytricha
Monanthotaxis filipes
Monanthotaxis sterilis
Monanthotaxis pellegrinii
Monanthotaxis pynaertii
Monanthotaxis letouzeyi
Monanthotaxis aff. letouzeyi
Monanthotaxis wieringae
Monanthotaxis cauliflora
Monanthotaxis aff. bidaultii
Monanthotaxis bidaultii
Monanthotaxis bicornis
Monanthotaxis malacophylla
Monanthotaxis submontana
Monanthotaxis quasilanceolata
Monanthotaxis gracilis
Monanthotaxis montana
Monanthotaxis glaucifolia
Monanthotaxis enghiana
Monanthotaxis velutina
Monanthotaxis hirsuta
Monanthotaxis obovata
Friesodielsia borneensis
Desmos dumosus
Dasymaschalon dasymaschalum
Toussaintia orientalis
Sphaerocoryne gracilis
Mitrella kentii
Uvaria scabrida
Uvaria afzelii

Fig. S1

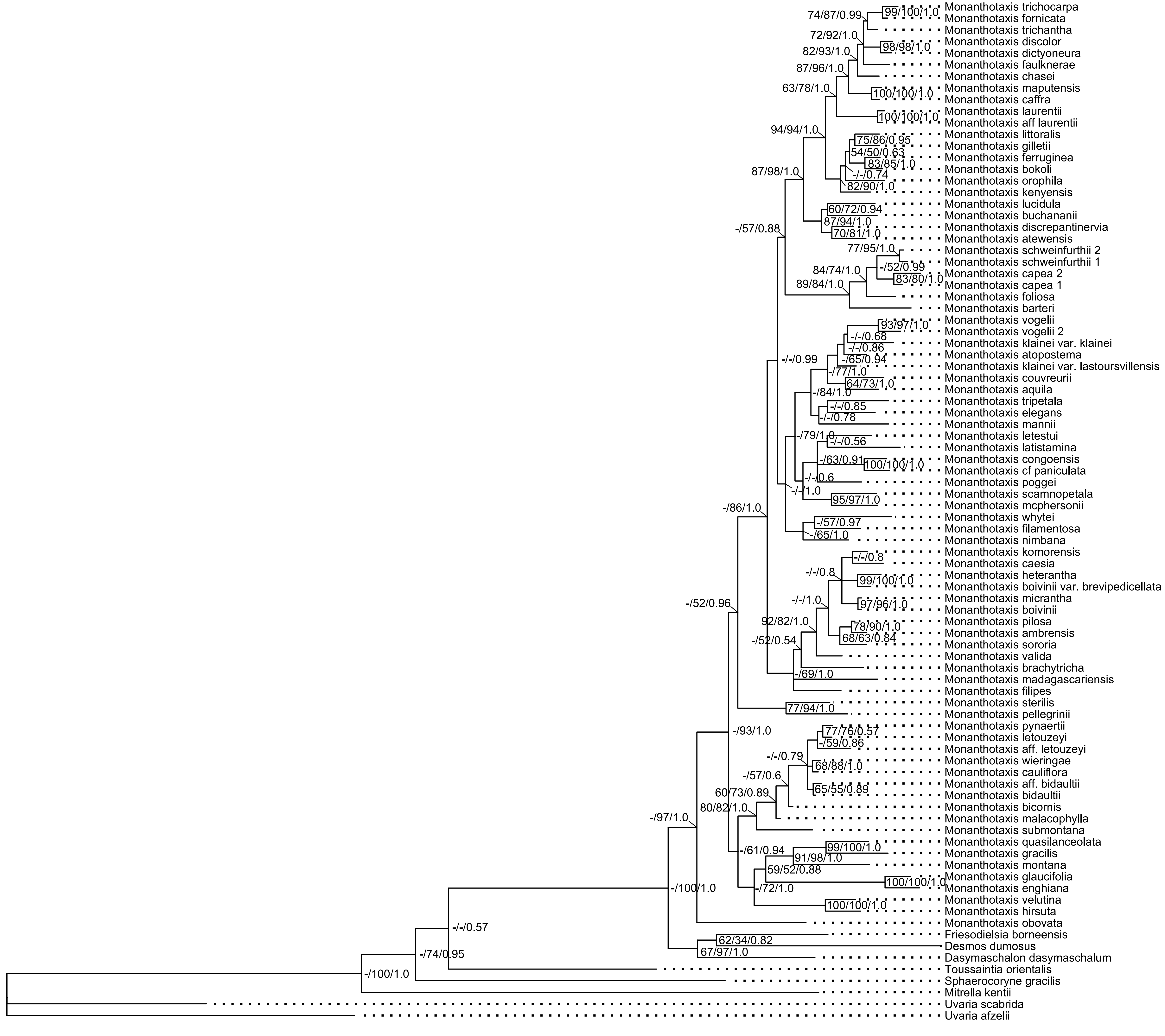

Monanthotaxis trichocarpa
Monanthotaxis fornicata
Monanthotaxis trichantha
Monanthotaxis discolor
Monanthotaxis dictyoneura
Monanthotaxis faulknerae
Monanthotaxis chasei
Monanthotaxis maputensis
Monanthotaxis caffra
Monanthotaxis laurentii
Monanthotaxis aff laurentii
Monanthotaxis littoralis
Monanthotaxis gilletii
Monanthotaxis ferruginea
Monanthotaxis bokoli
Monanthotaxis orophila
Monanthotaxis kenyensis
Monanthotaxis lucidula
Monanthotaxis buchananii
Monanthotaxis discrepantinervia
Monanthotaxis atewensis
Monanthotaxis schweinfurthii 2
Monanthotaxis schweinfurthii 1
Monanthotaxis capea 2
Monanthotaxis capea 1
Monanthotaxis foliosa
Monanthotaxis barteri
Monanthotaxis vogelii
Monanthotaxis vogelii 2
Monanthotaxis klainei var. klainei
Monanthotaxis atopostema
Monanthotaxis klainei var. lastoursvillensis
Monanthotaxis couvreurii
Monanthotaxis aquila
Monanthotaxis tripetala
Monanthotaxis elegans
Monanthotaxis mannii
Monanthotaxis letestui
Monanthotaxis latistamina
Monanthotaxis congoensis
Monanthotaxis cf paniculata
Monanthotaxis poggei
Monanthotaxis scamnopetala
Monanthotaxis mcphersonii
Monanthotaxis whytei
Monanthotaxis filamentosa
Monanthotaxis nimbana
Monanthotaxis komorensis
Monanthotaxis caesia
Monanthotaxis heterantha
Monanthotaxis boivinii var. brevipedicellata
Monanthotaxis micrantha
Monanthotaxis boivinii
Monanthotaxis pilosa
Monanthotaxis ambrensis
Monanthotaxis sororia
Monanthotaxis valida
Monanthotaxis brachytricha
Monanthotaxis madagascariensis
Monanthotaxis filipes
Monanthotaxis sterilis
Monanthotaxis pellegrinii
Monanthotaxis pynaertii
Monanthotaxis letouzeyi
Monanthotaxis aff. letouzeyi
Monanthotaxis wieringae
Monanthotaxis cauliflora
Monanthotaxis aff. bidaultii
Monanthotaxis bidaultii
Monanthotaxis bicornis
Monanthotaxis malacophylla
Monanthotaxis submontana
Monanthotaxis quasilanceolata
Monanthotaxis gracilis
Monanthotaxis montana
Monanthotaxis glaucifolia
Monanthotaxis enghiana
Monanthotaxis velutina
Monanthotaxis hirsuta
Monanthotaxis obovata
Friesodielsia borneensis
Desmos dumosus
Dasymaschalon dasymaschalum
Toussaintia orientalis
Sphaerocoryne gracilis
Mitrella kentii
Uvaria scabrida
Uvaria afzelii
99/100/1.0
74/87/0.99
72/92/1.0
98/98/1.0
82/93/1.0
87/96/1.0
100/100/1.0
63/78/1.0
100/100/1.0
94/94/1.0
75/86/0.95
54/50/0.63
83/85/1.0
-/-/0.74
82/90/1.0
87/98/1.0
60/72/0.94
87/94/1.0
70/81/1.0
-/57/0.88
77/95/1.0
-/52/0.99
84/74/1.0
83/80/1.0
89/84/1.0
93/97/1.0
-/-/0.68
-/-/0.86
-/65/0.94
-/-/0.99
-/77/1.0
64/73/1.0
-/84/1.0
-/-/0.85
-/-/0.78
-/79/1.0
-/-/0.56
-/63/0.91
100/100/1.0
-/-/0.6
-/-/1.0
95/97/1.0
-/86/1.0
-/57/0.97
-/65/1.0
-/-/0.8
-/-/0.8
99/100/1.0
-/-/1.0
97/96/1.0
-/52/0.96
92/82/1.0
78/90/1.0
68/63/0.84
-/52/0.54
-/69/1.0
77/94/1.0
-/93/1.0
77/76/0.57
-/59/0.86
-/-/0.79
68/88/1.0
-/57/0.6
65/55/0.89
60/73/0.89
-/97/1.0
80/82/1.0
99/100/1.0
-/61/0.94
91/98/1.0
59/52/0.88
100/100/1.0
-/100/1.0
-/72/1.0
100/100/1.0
-/-/0.57
62/34/0.82
-/74/0.95
67/97/1.0
-/100/1.0

Fig. S2

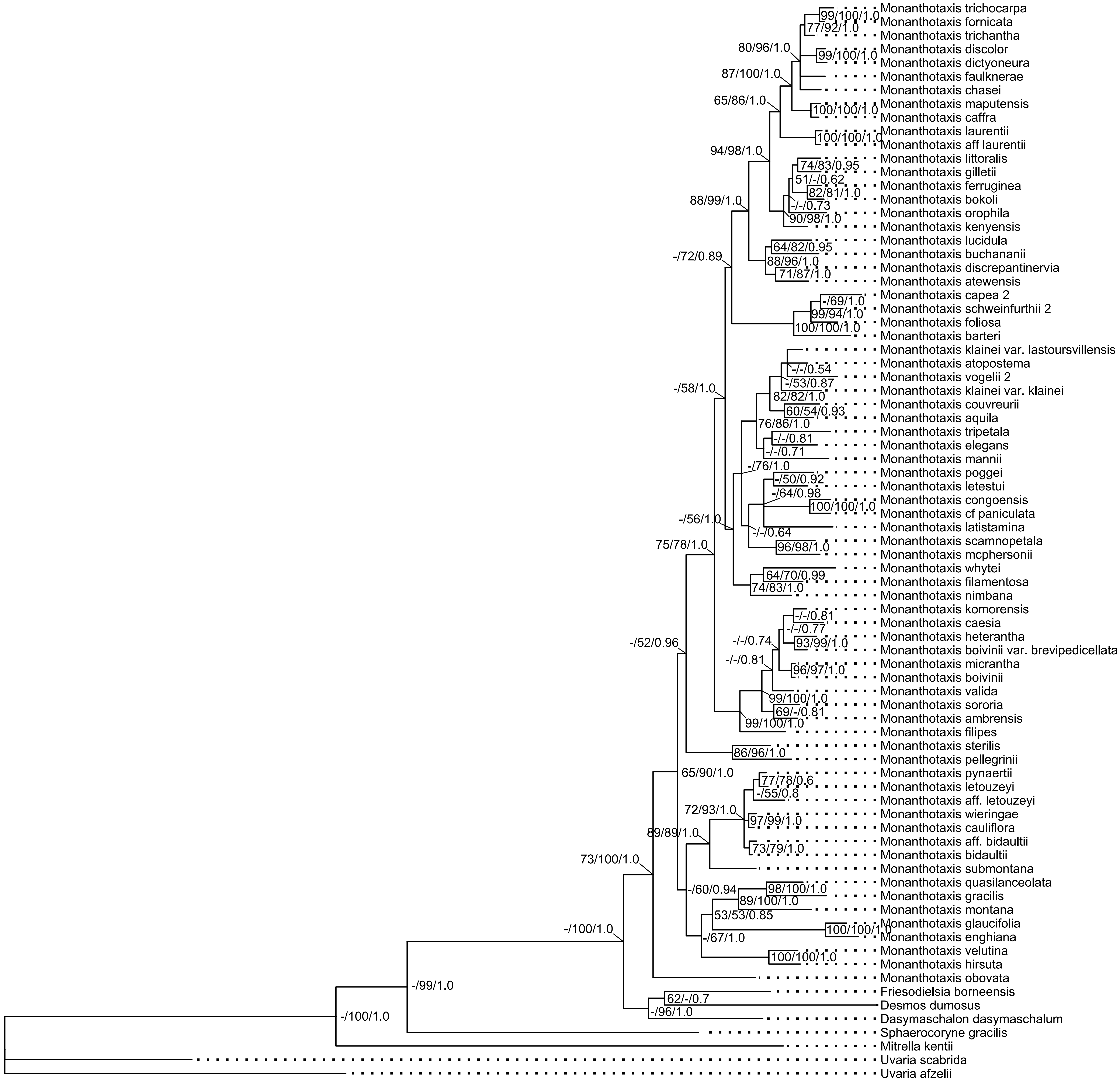
Monanthotaxis trichocarpa
Monanthotaxis fornicata
Monanthotaxis trichantha
Monanthotaxis discolor
Monanthotaxis dictyoneura
Monanthotaxis faulknerae
Monanthotaxis chasei
Monanthotaxis maputensis
Monanthotaxis caffra
Monanthotaxis laurentii
Monanthotaxis aff laurentii
Monanthotaxis littoralis
Monanthotaxis gilletii
Monanthotaxis ferruginea
Monanthotaxis bokoli
Monanthotaxis orophila
Monanthotaxis kenyensis
Monanthotaxis lucidula
Monanthotaxis buchananii
Monanthotaxis discrepantinervia
Monanthotaxis atewensis
Monanthotaxis capea 2
Monanthotaxis schweinfurthii 2
Monanthotaxis foliosa
Monanthotaxis barteri
Monanthotaxis klainei var. lastoursvillensis
Monanthotaxis atopostema
Monanthotaxis vogelii 2
Monanthotaxis klainei var. klainei
Monanthotaxis couvreurii
Monanthotaxis aquila
Monanthotaxis tripetala
Monanthotaxis elegans
Monanthotaxis mannii
Monanthotaxis poggei
Monanthotaxis letestui
Monanthotaxis congoensis
Monanthotaxis cf paniculata
Monanthotaxis latistamina
Monanthotaxis scamnopetala
Monanthotaxis mcphersonii
Monanthotaxis whytei
Monanthotaxis filamentosa
Monanthotaxis nimbana
Monanthotaxis komorensis
Monanthotaxis caesia
Monanthotaxis heterantha
Monanthotaxis boivinii var. brevipedicellata
Monanthotaxis micrantha
Monanthotaxis boivinii
Monanthotaxis valida
Monanthotaxis sororia
Monanthotaxis ambrensis
Monanthotaxis filipes
Monanthotaxis sterilis
Monanthotaxis pellegrinii
Monanthotaxis pynaertii
Monanthotaxis letouzeyi
Monanthotaxis aff. letouzeyi
Monanthotaxis wieringae
Monanthotaxis cauliflora
Monanthotaxis aff. bidaultii
Monanthotaxis bidaultii
Monanthotaxis submontana
Monanthotaxis quasilanceolata
Monanthotaxis gracilis
Monanthotaxis montana
Monanthotaxis glaucifolia
Monanthotaxis enghiana
Monanthotaxis velutina
Monanthotaxis hirsuta
Monanthotaxis obovata
Friesodielsia borneensis
Desmos dumosus
Dasymaschalon dasymaschalum
Sphaerocoryne gracilis
Mitrella kentii
Uvaria scabrida
Uvaria afzelii
99/100/1.0
77/92/1.0
80/96/1.0
99/100/1.0
87/100/1.0
65/86/1.0
100/100/1.0
100/100/1.0
94/98/1.0
74/83/0.95
51/-/0.62
82/81/1.0
-/-/0.73
90/98/1.0
88/99/1.0
64/82/0.95
88/96/1.0
71/87/1.0
-/72/0.89
-/69/1.0
99/94/1.0
100/100/1.0
-/-/0.54
-/53/0.87
-/58/1.0
82/82/1.0
60/54/0.93
76/86/1.0
-/-/0.81
-/-/0.71
-/76/1.0
-/50/0.92
-/64/0.98
100/100/1.0
-/56/1.0
-/-/0.64
96/98/1.0
75/78/1.0
64/70/0.99
74/83/1.0
-/-/0.81
-/-/0.77
-/-/0.74
93/99/1.0
-/-/0.81
96/97/1.0
-/52/0.96
99/100/1.0
69/-/0.81
99/100/1.0
86/96/1.0
65/90/1.0
77/78/0.6
-/55/0.8
72/93/1.0
97/99/1.0
89/89/1.0
73/79/1.0
73/100/1.0
-/60/0.94
98/100/1.0
89/100/1.0
53/53/0.85
100/100/1.0
-/100/1.0
-/67/1.0
100/100/1.0
-/99/1.0
62/-/0.7
-/96/1.0
-/100/1.0

Fig. S3

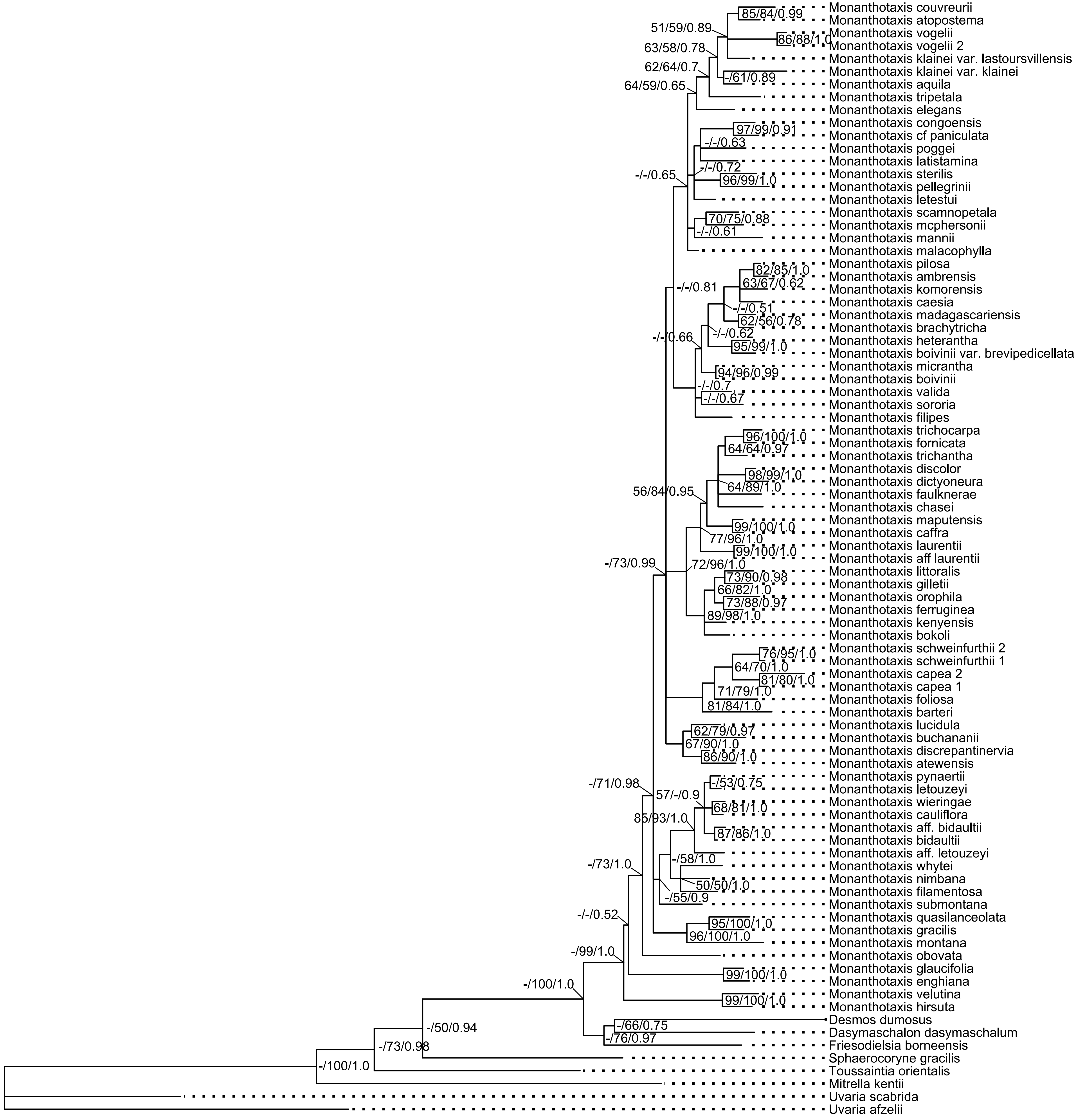
Monanthotaxis couvreurii
Monanthotaxis atopostema
Monanthotaxis vogelii
Monanthotaxis vogelii 2
Monanthotaxis klainei var. lastoursvillensis
Monanthotaxis klainei var. klainei
Monanthotaxis aquila
Monanthotaxis tripetala
Monanthotaxis elegans
Monanthotaxis congoensis
Monanthotaxis cf paniculata
Monanthotaxis poggei
Monanthotaxis latistamina
Monanthotaxis sterilis
Monanthotaxis pellegrinii
Monanthotaxis letestui
Monanthotaxis scamnopetala
Monanthotaxis mcphersonii
Monanthotaxis mannii
Monanthotaxis malacophylla
Monanthotaxis pilosa
Monanthotaxis ambrensis
Monanthotaxis komorensis
Monanthotaxis caesia
Monanthotaxis madagascariensis
Monanthotaxis brachytricha
Monanthotaxis heterantha
Monanthotaxis boivinii var. brevipedicellata
Monanthotaxis micrantha
Monanthotaxis boivinii
Monanthotaxis valida
Monanthotaxis sororia
Monanthotaxis filipes
Monanthotaxis trichocarpa
Monanthotaxis fornicata
Monanthotaxis trichantha
Monanthotaxis discolor
Monanthotaxis dictyoneura
Monanthotaxis faulknerae
Monanthotaxis chasei
Monanthotaxis maputensis
Monanthotaxis caffra
Monanthotaxis laurentii
Monanthotaxis aff laurentii
Monanthotaxis littoralis
Monanthotaxis gilletii
Monanthotaxis orophila
Monanthotaxis ferruginea
Monanthotaxis kenyensis
Monanthotaxis bokoli
Monanthotaxis schweinfurthii 2
Monanthotaxis schweinfurthii 1
Monanthotaxis capea 2
Monanthotaxis capea 1
Monanthotaxis foliosa
Monanthotaxis barteri
Monanthotaxis lucidula
Monanthotaxis buchananii
Monanthotaxis discrepantinervia
Monanthotaxis atewensis
Monanthotaxis pynaertii
Monanthotaxis letouzeyi
Monanthotaxis wieringae
Monanthotaxis cauliflora
Monanthotaxis aff. bidaultii
Monanthotaxis bidaultii
Monanthotaxis aff. letouzeyi
Monanthotaxis whytei
Monanthotaxis nimbana
Monanthotaxis filamentosa
Monanthotaxis submontana
Monanthotaxis quasilanceolata
Monanthotaxis gracilis
Monanthotaxis montana
Monanthotaxis obovata
Monanthotaxis glaucifolia
Monanthotaxis enghiana
Monanthotaxis velutina
Monanthotaxis hirsuta
Desmos dumosus
Dasymaschalon dasymaschalum
Friesodielsia borneensis
Sphaerocoryne gracilis
Toussaintia orientalis
Mitrella kentii
Uvaria scabrida
Uvaria afzelii
85/84/0.99
51/59/0.89
86/88/1.0
63/58/0.78
62/64/0.7
-/61/0.89
64/59/0.65
97/99/0.91
-/-/0.63
-/-/0.65
-/-/0.72
96/99/1.0
70/75/0.88
-/-/0.61
82/85/1.0
-/-/0.81
63/67/0.62
-/-/0.51
62/56/0.78
-/-/0.66
-/-/0.62
95/99/1.0
94/96/0.99
-/-/0.7
-/-/0.67
96/100/1.0
64/64/0.97
98/99/1.0
64/89/1.0
56/84/0.95
99/100/1.0
77/96/1.0
99/100/1.0
-/73/0.99
72/96/1.0
73/90/0.98
66/82/1.0
73/88/0.97
89/98/1.0
76/95/1.0
64/70/1.0
81/80/1.0
71/79/1.0
81/84/1.0
62/79/0.97
67/90/1.0
86/90/1.0
-/53/0.75
-/71/0.98
57/-/0.9
68/81/1.0
85/93/1.0
87/86/1.0
-/58/1.0
-/73/1.0
50/50/1.0
-/55/0.9
95/100/1.0
-/-/0.52
96/100/1.0
-/99/1.0
99/100/1.0
-/100/1.0
99/100/1.0
-/66/0.75
-/50/0.94
-/76/0.97
-/73/0.98
-/100/1.0

Fig. S4

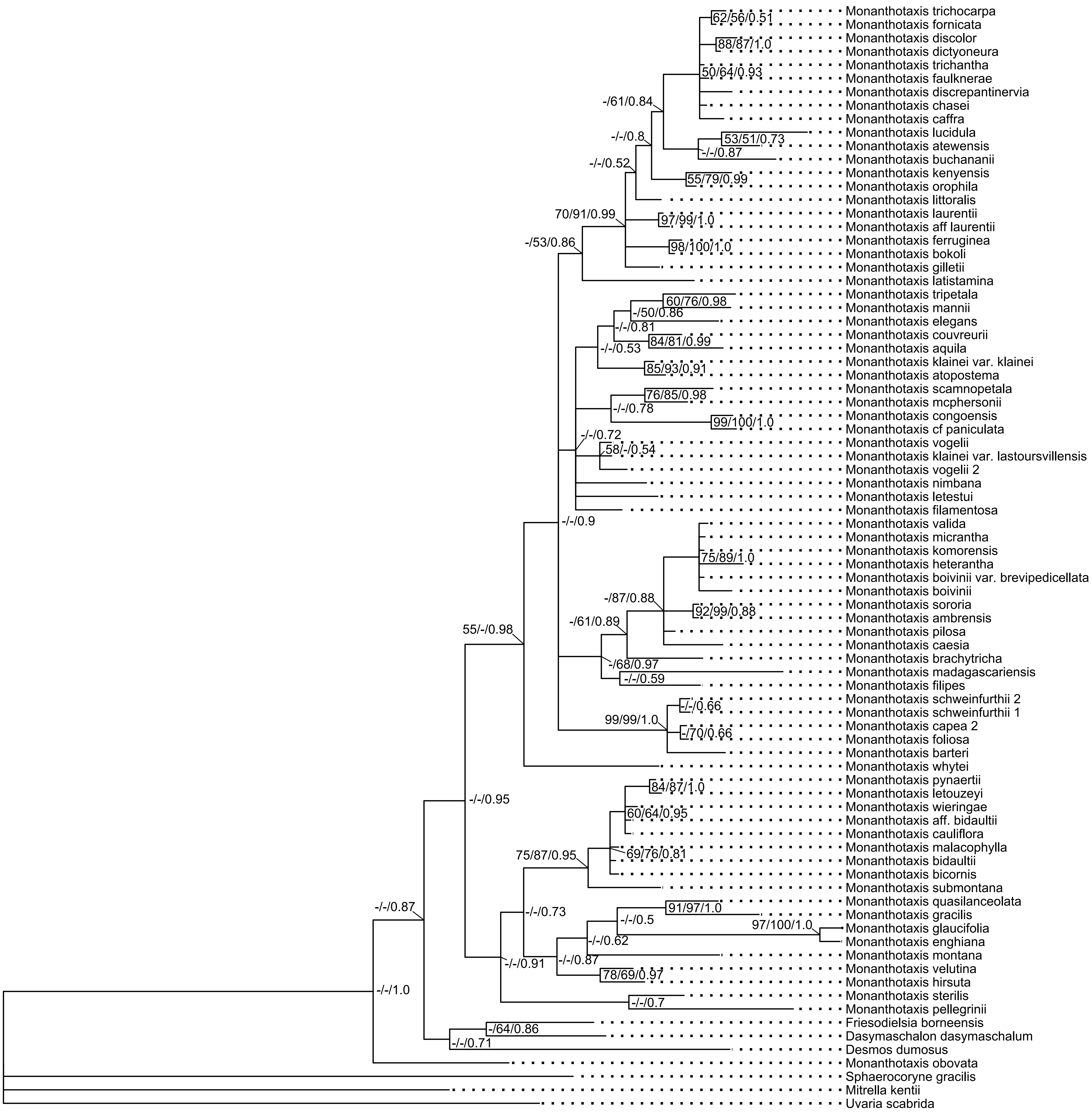
62/56/0.51
88/87/1.0
50/64/0.93
-/61/0.84
-/-/0.8
53/51/0.73
-/-/0.87
-/-/0.52
55/79/0.99
70/91/0.99
97/99/1.0
98/100/1.0
-/53/0.86
60/76/0.98
-/50/0.86
-/-/0.81
84/81/0.99
-/-/0.53
85/93/0.91
76/85/0.98
-/-/0.78
99/100/1.0
-/-/0.72
58/-/0.54
-/-/0.9
75/89/1.0
-/87/0.88
92/99/0.88
-/61/0.89
-/68/0.97
-/-/0.59
99/99/1.0
-/-/0.66
-/70/0.66
55/-/0.98
-/-/0.95
84/87/1.0
60/64/0.95
69/76/0.81
75/87/0.95
-/-/0.73
91/97/1.0
-/-/0.5
97/100/1.0
-/-/0.62
-/-/0.87
-/-/0.91
78/69/0.97
-/-/0.7
-/-/0.87
-/-/1.0
-/64/0.86
-/-/0.71
Monanthotaxis trichocarpa
Monanthotaxis fornicata
Monanthotaxis discolor
Monanthotaxis dictyoneura
Monanthotaxis trichantha
Monanthotaxis faulknerae
Monanthotaxis discrepantinervia
Monanthotaxis chasei
Monanthotaxis caffra
Monanthotaxis lucidula
Monanthotaxis atewensis
Monanthotaxis buchananii
Monanthotaxis kenyensis
Monanthotaxis orophila
Monanthotaxis littoralis
Monanthotaxis laurentii
Monanthotaxis aff laurentii
Monanthotaxis ferruginea
Monanthotaxis bokoli
Monanthotaxis gilletii
Monanthotaxis latistamina
Monanthotaxis tripetala
Monanthotaxis mannii
Monanthotaxis elegans
Monanthotaxis couvreurii
Monanthotaxis aquila
Monanthotaxis klainei var. klainei
Monanthotaxis atopostema
Monanthotaxis scamnopetala
Monanthotaxis mcphersonii
Monanthotaxis congoensis
Monanthotaxis cf paniculata
Monanthotaxis vogelii
Monanthotaxis klainei var. lastoursvillensis
Monanthotaxis vogelii 2
Monanthotaxis nimbana
Monanthotaxis letestui
Monanthotaxis filamentosa
Monanthotaxis valida
Monanthotaxis micrantha
Monanthotaxis komorensis
Monanthotaxis heterantha
Monanthotaxis boivinii var. brevipedicellata
Monanthotaxis boivinii
Monanthotaxis sororia
Monanthotaxis ambrensis
Monanthotaxis pilosa
Monanthotaxis caesia
Monanthotaxis brachytricha
Monanthotaxis madagascariensis
Monanthotaxis filipes
Monanthotaxis schweinfurthii 2
Monanthotaxis schweinfurthii 1
Monanthotaxis capea 2
Monanthotaxis foliosa
Monanthotaxis barteri
Monanthotaxis whytei
Monanthotaxis pynaertii
Monanthotaxis letouzeyi
Monanthotaxis wieringae
Monanthotaxis aff. bidaultii
Monanthotaxis cauliflora
Monanthotaxis malacophylla
Monanthotaxis bidaultii
Monanthotaxis bicornis
Monanthotaxis submontana
Monanthotaxis quasilanceolata
Monanthotaxis gracilis
Monanthotaxis glaucifolia
Monanthotaxis enghiana
Monanthotaxis montana
Monanthotaxis velutina
Monanthotaxis hirsuta
Monanthotaxis sterilis
Monanthotaxis pellegrinii
Friesodielsia borneensis
Dasymaschalon dasymaschalum
Desmos dumosus
Monanthotaxis obovata
Sphaerocoryne gracilis
Mitrella kentii
Uvaria scabrida

Fig. S5
